# Genomic and metabolomic insights into the antimicrobial and therapeutic potential of *Lactiplantibacillus plantarum* UTNGt28L isolated from Amazonian star apple

**DOI:** 10.3389/fmicb.2026.1771106

**Published:** 2026-02-18

**Authors:** Gabriela N. Tenea, Ioana C. Marinas, Gratiela Gradisteanu Pircalabioru, Jazmin Hidalgo, Mariana C. Chifiriuc, Mayte Noboa

**Affiliations:** 1 Biofood and Nutraceutics Research and Development Group, Faculty of Engineering in Agricultural and Environmental Sciences, Universidad Técnica del Norte, Ibarra, Ecuador; 2 Research Institute of the University of Bucharest—ICUB, University of Bucharest, Bucharest, Romania

**Keywords:** GC-MS metabolomics, *Lactiplantibacillus plantarum*, phenyllactic acid, postbiotics, probiotic safety, γ-aminobutyric acid

## Abstract

**Introduction:**

Probiotic candidates from underexplored ecological niches represent a valuable source of novel functional traits. This study reports, for the first time, the probiotic potential and safety profile of *Lactiplantibacillus plantarum* UTNGt28L (Gt28L), a strain isolated from the Amazonian star apple (*Chrysophyllum cainito*), using an integrated multi-omics approach to link genomic features with functional bioactivity.

**Methods:**

Whole-genome sequencing was performed followed by functional annotation, phylogenetic analysis, and biosynthetic gene cluster (BGC) prediction. Untargeted intracellular metabolomic profiling (Met-Int) was conducted using gas chromatography coupled with quadrupole time-of-flight mass spectrometry (GC/MS-QTOF). *In silico* analyses were applied to assess antimicrobial potential, virulence determinants, antibiotic resistance genes (ARGs), and ADME-Tox (absorption, distribution, metabolism, excretion, and toxicity) properties. Additionally, extracellular metabolites (Met-Ext) from culture supernatants were characterized for chemical composition, antioxidant activity, and total polyphenol content using in vitro assays.

**Results:**

Genomic analysis revealed a stable and biosafe genome (3.23 Mb) devoid of mobile virulence factors and ARGs, while encoding multiple class IIb plantaricins consistent with broad-spectrum antimicrobial activity. Metabolomic profiling identified several bioactive compounds, including phenyllactic acid, γ-aminobutyric acid, 3-(4-hydroxyphenyl)lactic acid, and benzoic acid. In vitro and *in silico* evaluations supported the strain’s safety, favorable ADME characteristics, and functional antioxidant and antimicrobial properties, establishing a strong correlation between genomic potential and observed bioactivity.

**Conclusion:**

*L. plantarum* UTNGt28L exhibits a robust safety profile and multifunctional probiotic traits supported by integrated genomic and metabolomic evidence. These findings position Gt28L as a promising candidate for probiotic and postbiotic applications in food, health, and pharmaceutical biotechnology, particularly leveraging the biodiversity of Amazonian ecosystems.

## 1 Introduction

*Lactiplantibacillus plantarum* (formerly *Lactobacillus plantarum*) is a diverse and adaptable species within the lactic acid bacteria (LAB), widely recognized for its robust probiotic potential and versatile role in food fermentation (Behera et al., 2018; Gizachew and Engidawork, 2024). *L. plantarum* exhibits a remarkably large and flexible genome compared to other LAB species, ranging from approximately 3.0–3.5 Mb, with a high GC content and numerous strain-specific genes associated with carbohydrate metabolism, stress responses, and host interaction (Carpi et al., 2022). These features enable *L. plantarum* to thrive in diverse ecological niches, including fermented foods, the human gastrointestinal tract, and plant surfaces. This remarkable genomic and ecological plasticity correlates with the wide-ranging production of bioactive metabolites, including organic acids and antimicrobial metabolites, which contribute to its probiotic functions, including pathogen inhibition, immunomodulation, and reinforcement of the gut barrier (Marco et al., 2006; Shah et al., 2024). The composition in health-promoting and antimicrobial metabolites is often strain-specific, requiring the isolation and characterization of individual strains to identify appropriate candidates with optimal functional traits for targeted probiotic or therapeutic applications (Kim et al., 2021; Carpi et al., 2022).

Previous work has highlighted the importance of ecological origin in shaping LAB strains’ genomic and phenotypic profiles (Tenea, 2022). By investigating the microbial communities of Amazonian fruits, we identified LAB strains with distinct antimicrobial and probiotic potential (Benavides et al., 2016). Environmental pressures, such as nutrient availability, competition, and host interactions, drive strain-level diversity through mechanisms like horizontal gene transfer (HGT), specialized metabolic adaptation, and dynamic stress responses (Tenea and Ortega, 2021; Carpi et al., 2022). Comparative genomic studies of *L. plantarum* isolates from fruits have revealed niche-specific genetic adaptations, including genes associated with acid resistance, oxidative stress, and the production of health-promoting metabolites (Tenea, 2022). Following this work, a targeted microbiological survey was conducted in the Amazonian region to expand the collection of LAB isolates from underexplored fruit sources. As part of this expedition, samples were collected from *Chrysophyllum cainito* (star apple), from the *Sapotaceae* family. Beyond its nutritional appeal, the fruit has been widely used in traditional medicine for its anti-inflammatory, antidiabetic, and antioxidant properties. These therapeutic effects are largely attributed to its diverse phytochemical profile, including flavonoids, tannins, and saponins, which have demonstrated *in vitro* and *in vivo* antimicrobial, hepatoprotective, and antihyperglycemic activities (Nguyen et al., 2019; Floriano et al., 2020; Akinmoladun et al., 2020). The bioactive potential of *C. cainito* and other Amazonian fruits extends beyond plant metabolites, as these complex ecological niches also host a rich diversity of microbial communities. Subsequent microbiological analyses led to the isolation of multiple LAB strains, several of which demonstrated significant *in vitro* antimicrobial activity. These findings reinforce the role of tropical fruits as unique ecological niches for the isolation of novel LAB with strain-specific genomic and functional attributes, thereby contributing to the discovery of microbial resources with potential applications in food, health, and biotechnology (Benavides et al., 2016). Among several isolates, the strain *L. plantarum* Gt28L was selected for its significant antimicrobial activity and distinctive genetic and metabolic profile shaped by its unique ecological origin. Considering the strong influence of environmental pressures on the evolution of microbial genomes and metabolomes (Smid and Lacroix, 2013), comprehensive genomic and metabolomic analyses of Gt28L are crucial to elucidate its adaptive mechanisms and to evaluate its probiotic and therapeutic potential. The objective of this study was to comprehensively characterize the Gt28L isolate using an integrated multi-omics approach. Genomic, metabolomic, and functional analyses were employed to elucidate its genetic architecture, biosynthetic potential, metabolic profile, and safety-related features. Specifically, genome-based analyses were used to assess evolutionary relationships, biosynthetic gene clusters, and antimicrobial resistance determinants, while untargeted metabolomics enabled the identification of intracellular and extracellular bioactive metabolites. In parallel, *in vitro* assays were conducted to evaluate antioxidant activity, hemocompatibility, epithelial adhesion, and immunological relevance. Through this integrative framework, the study aimed to establish the biotechnological and functional potential of Gt28L as a source of bioactive metabolites and probiotic-related traits.

## 2 Materials and methods

### 2.1 Bacterial strain

**FIGURE 1:**
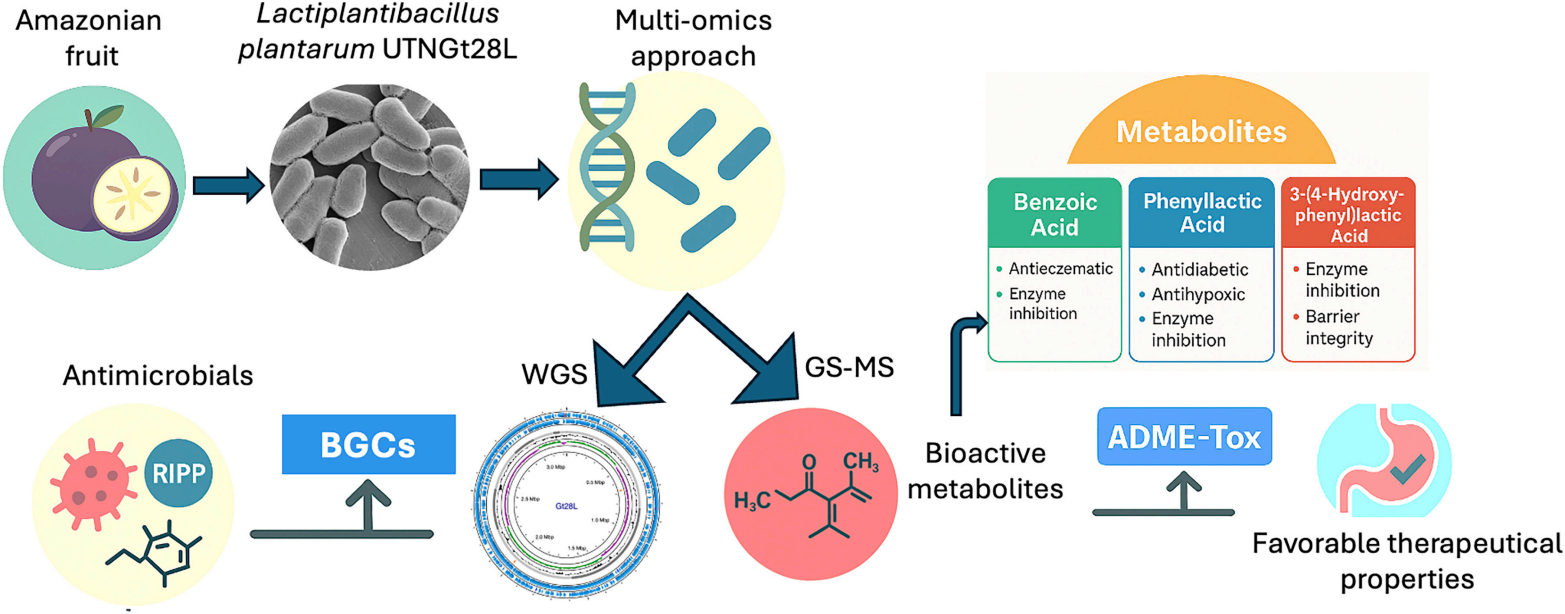
Schematic overview of the multi-omics strategy used to characterize *Lactiplantibacillus plantarum* UTNGt28L isolated from an Amazonian fruit. Whole-genome sequencing (WGS) enabled the identification of biosynthetic gene clusters (BGCs) and antimicrobial-related features, while GC–MS analysis revealed bioactive metabolites. Detected compounds, including benzoic acid, phenyllactic acid, and 3-(4-hydroxyphenyl) lactic acid, were associated with antimicrobial and health-related activities. *In silico* Absorption, Distribution, Metabolism, Excretion, and Toxicity (ADME–Tox) analysis indicated favorable therapeutic properties of the identified metabolites.

The isolate Gt28L was obtained from star apple fruits (*C. cainito*) collected in July 2016 from the Amazon rainforest in the Sucumbíos Province, Ecuador, following the protocol described by Tenea and Ortega (2021). The strain was routinely cultured in de Man, Rogosa and Sharpe (MRS) broth (Difco, Detroit, MI, United States) and preserved at -80°C in MRS supplemented with 20% (v/v) glycerol for long-term storage. The microorganism has been deposited in the CCMBIOGEM Laboratory Culture Collection and is available upon reasonable request, in accordance with institutional policies. The schematic overview of the bacterial characterization is depicted in Figure 1.

### 2.2 Genome sequencing, gene prediction, and functional annotation

Whole-genome *de novo* sequencing and assembly of the Gt28L strain were performed following the methodology previously described by Tenea and Ortega (2021), using sequencing services provided by Macrogen Inc. (Seoul, Republic of Korea). Genomic DNA libraries were prepared via random fragmentation followed by adapter ligation. Raw sequence quality was evaluated using FastQC v0.11.5, and adapter removal and quality trimming were carried out with Trimmomatic v0.36. High-quality reads were further assessed for base quality distribution, GC content, and read abundance prior to assembly. Genome assembly was conducted using SPAdes v3.15.5 with multiple k-mer sizes, supported by k-mer frequency profiling using Jellyfish v2.2.10. The *de Bruijn* graph-based assembly generated 14,106,374 reads, achieving an average sequencing coverage of 212 × , a GC content of 44.54%, and a Q30 score of 92.18%. Assembly validation through read mapping and BUSCO analysis resulted in 12 contigs totaling 3,231,781 bp, with an N50 of 662,366 bp. BUSCO assessment indicated a high level of genome completeness (99.19% complete, and 0.81% duplicated genes). Additional assembly statistics are provided in Supplementary Tables 1–3. Structural and functional annotation of the Gt28L genome was conducted using a previously established pipeline (Tenea and Ortega, 2021), with PROKKA (Seemann, 2014) for gene prediction and InterProScan and EggNOG (Huerta-Cepas et al., 2019) for functional categorization. Annotations were refined using the Global Catalog of Microorganisms pipeline^1^ (Shi et al., 2021), referencing key databases including SwissProt, MetaCyc (Caspi et al., 2020), VFDB (virulence factors database), PHI (Pathogen-Host Interaction database), KEGG (Kyoto Encyclopedia of Genes and Genomes), and COG (Clusters of Orthologous Groups of proteins). Moreover, ARGs were identified using CARD and the RGI tool (Jia et al., 2017; Zankari et al., 2012), while ResFinder 4.1 (Bortolaia et al., 2020) was employed to detect acquired resistance and chromosomal mutations (≥ 90% identity, ≥ 60% length). The pathogenic potential was assessed using PathogenFinder (Cosentino et al., 2013).

### 2.3 Taxonomy and phylogenetic relationship

Species identification was performed using BLAST against the NCBI NT database, with ANI (Average Nucleotide Identity) values ≥ 95–96% confirming species identity (Richter and Rosselló-Móra, 2009). A circular genome map was generated using Proksee server (Grant et al., 2023). For phylogenetic analysis, the genome FASTA file of strain Gt28L was submitted to the gcType platform, (see text footnote 1) which integrates the Type Strain Genome Database (Shi et al., 2021). The closest type strains were identified by comparing the Gt28L genome against all available type strain genomes in the TYGS database using the Mash algorithm (Meier-Kolthoff and Göker, 2019). Genomes with the smallest Mash distances were automatically selected for downstream analyses. Intergenomic distances were subsequently calculated using the Genome BLAST Distance Phylogeny (GBDP) method, applying the coverage algorithm and the recommended distance formula to ensure accurate phylogenetic resolution (Meier-Kolthoff et al., 2013). Species-level identification was further refined using multiple genome similarity metrics, including ANIb (Richter and Rosselló-Móra, 2009), FastANI, OrthoANIb, and OrthoANIu, to assess nucleotide identity between the query and reference genomes. For phylogenetic reconstruction, 16S rRNA gene sequences were aligned using MAFFT or MUSCLE, and phylogenetic trees were inferred using MEGA, FastTree, and RAxML. In addition, 56 conserved single-copy marker genes were extracted from the selected genomes to construct a robust phylogenomic tree.

### 2.4 Biosynthetic gene clusters and secondary metabolites prediction

FASTA-formatted contigs were subjected to analysis with BAGEL4 to identify BGCs involved in the production of antimicrobial peptides, including bacteriocins (de Jong et al., 2006). To complement this, antiSMASH v6.0.1 (Antibiotics and Secondary Metabolite Analysis Shell) was employed for the genome-wide prediction of BGCs for secondary metabolite, with a particular focus on clusters typical of anaerobic bacteria (Blin et al., 2021).

### 2.5 Pangenome analysis

Pangenome analysis was performed using Roary (v1.007001)^2^ (Page et al., 2015), with MAFFT v7.427 (Katoh and Standley, 2013) for sequence alignment. Protein-coding genes were clustered into core (hardcore and softcore) and accessory (shell and cloud) genomes based on their presence across the analyzed strains. Genomic data for *Lactobacillus* (*n* = 9) were retrieved from NCBI (Supplementary Table 4) selected based on their origin from plant- or fruit-derived (vegetal) substrates and the availability of high-quality assemblies with complete metadata, to ensure ecological relevance and robust comparative analyses. Gene clusters were classified as core (> 99%), soft core (95–99%), shell (15–95%), or cloud (< 15%), providing insights into conserved and variable genomic content and adaptive potential.

### 2.6 Chemical characterization of Gt28L intracellular metabolite extract by GC/MS-QTOF

#### 2.6.1 Met-Int extraction

To extract intracellular metabolites, bacterial cultures were grown as three independent biological replicates in de Man, Rogosa, and Sharpe (MRS) broth and incubated at 37°C for 24 h. Cultures were centrifuged at 13,000 × g for 30 min at 4°C, and the resulting pellets were resuspended in methanol: water (4:1, v/v; HPLC–MS grade). Cells were subjected to three freeze–thaw cycles using liquid nitrogen, followed by incubation at 35°C for 5 min, vigorous vortexing, and sonication at 20 kHz for 2 min on ice. The suspensions were then centrifuged at 13,000 × g for 10 min at 4°C to recover the supernatants containing intracellular metabolite extracts (Met-Int). Each biological replicate was processed in technical triplicate. The extracts were lyophilized and stored until further analysis.

#### 2.6.2 Sample preparation for GC/MS-QTOF

Met-Int extracts were reconstituted in 500 μL of methanol, vortexed for 10 min, subjected to ultrasonic extraction for 10 min, and vortexed again for 5 min. The resulting suspensions were filtered through 0.22 μm PTFE membranes prior to derivatization. For chemical derivatization, 30 μL of each filtrate were evaporated to dryness in a SpeedVac concentrator at 35°C for 1 h. Dried residues were treated with 20 μL of O-methoxyamine hydrochloride in pyridine (15 mg/mL), vortexed at 3,200 rpm for 5 min, and incubated in the dark at room temperature for 16 h. Silylation was then carried out by adding 20 μL of N,O-bis(trimethylsilyl)trifluoroacetamide (BSTFA) containing 1% trimethylchlorosilane (TMCS), followed by vortexing for 5 min and incubation at 70°C for 1 h. After cooling to room temperature for 30 min, 140 μL of methyl stearate in heptane (5 mg/L) were added as an internal standard, and the mixture was vortexed for 5 min at 3,200 rpm prior to GC–MS analysis.

#### 2.6.3 Data acquisition

Data acquisition was performed using an Agilent Technologies 7890B gas chromatograph coupled to an Agilent Technologies GC/Q-TOF 7250 time-of-flight mass selective detector, equipped with a split/splitless injection port (250°C, split ratio 50) and an Agilent Technologies 7693A autosampler. The electron ionization (EI) source was operated at 70 eV. An Agilent Technologies J&W HP-5MS column (30 m, 0.25 mm, 0.25 μm) was used, with helium as the carrier gas at a constant flow of 0.7 mL/min. The oven temperature was programmed from 60°C (1 min) at 10°C/min to 325°C (10 min). The transfer line temperature to the detector, the ion source filament, and the quadrupole were maintained at 280, 230, and 150°C, respectively. Mass spectrometry detection was performed over the range of 50–600 m/z at a rate of 5 spectra/s.

#### 2.6.4 Metabolite identification

The deconvolution of the data and metabolite identification were performed using the software Agilent MassHunter Unknowns Analysis B.10.00. Identification was carried out by searching two specific libraries: the “Fiehn GC-MS Metabolomics RTL Library” version 2011 and the NIST Mass Spectral Reference Library (National Institute of Standards and Technology) version 2017. The matching criteria included retention time, mass spectrum, and/or retention indices (RI) of FAMES (fatty acid methyl esters) or alkanes C7 to C40. Metabolites detected by GC–MS were annotated and linked to metabolic pathways using MetaboAnalyst Pathway Analysis 6.0. This platform integrates data from sources including HMDB, PubChem, and KEGG to translate compound identifiers and infer their biological functions (Pang et al., 2024; Kanehisa et al., 2024).

#### 2.6.5 Prediction of pharmacological potential of met-int derived from Gt28L *in silico*

The pharmacokinetic properties related to Absorption, Distribution, Metabolism, and Excretion (ADME) of selected metabolites were predicted *in silico* using the SwissADME platform.^3^ Chemical structures, provided in SMILES format, were submitted to evaluate drug-likeness and ADME-related descriptors. Acute toxicity and median lethal dose (LD50) were estimated using the ProTox 3.0 tool.^4^ Potential pharmacological activities of the metabolites were explored using the PASS online server,^5^ which assigns probabilities of activity (Pa) or inactivity (Pi) based on structure–activity relationships derived from curated training datasets (Martiz et al., 2023; Kumari et al., 2023). These PASS outputs were used as hypothesis-generating indicators to prioritize putative biological activities for further experimental validation, rather than as confirmatory evidence of pharmacological function.

### 2.7 External metabolites characterization

#### 2.7.1 Extraction

Supernatant was obtained from overnight cultures of the Gt28L strain grown in MRS broth at 37°C, following the method of Garzón et al. (2017). After culture, the supernatant was passed through a 0.22 μm sterile syringe filter (Cat. #STF020025H, Chemlab Group, Washington, DC, United States) to eliminate residual bacterial cells and particulate matter. The filtered CFS was then neutralized to pH 6.0 to eliminate the effect of organic acids, lyophilized, and stored for use in subsequent analytical assays.

#### 2.7.2 Attenuated total reflectance–fourier transform infrared spectroscopy assay

The FTIR spectra of all freeze-dried substances were obtained using a Cary 630 FTIR Spectrometer in ATR mode and the Agilent MicroLab Software FTIR System (Agilent Technologies, Inc., United States). The results were obtained throughout a spectral range of 4,000–650 cm^–1^, using 400 scans at a resolution of 4 cm^–1^, under room temperature and air. Freeze-dried MRS broth served as a control (Marinas et al., 2023).

#### 2.7.3 *In vitro* antioxidant activity

##### 2.7.3.1 2 ,2-diphenyl-1-picrylhydrazyl radical scavenging assay

The DPPH radical scavenging activity was assessed by mixing 200 μL of a 0.3 mM DPPH methanolic solution with 200 μL of Met-Ext or MRS-broth samples at various dilutions. The mixtures were incubated at room temperature for 10 min, followed by centrifugation at 3,000 × g for 20 min. Absorbance was measured at 517 nm using a UV–VIS spectrophotometer (Multiskan FC, Thermo Scientific). The percentage of DPPH inhibition was calculated using the following formula: DPPH inhibition (%) = A-B/A × 100; where A represents the absorbance of the solvent control (control—DPPH) and B denotes the absorbance of the sample. The half-maximal inhibitory concentration (IC50) was determined by linear regression of concentration vs. percentage inhibition (Madhu et al., 2013). Further analysis was performed using a four-parameter variable slope model [log (inhibitor) vs. response] in GraphPad Prism v10.0 (GraphPad Software, San Diego, CA, United States).

##### 2.7.3.2 Ferric reducing antioxidant power assay

The FRAP assay was conducted according to the method of Click or tap here to enter text. with minor modifications. Briefly, 285 μL of freshly prepared FRAP reagent was mixed with 15 μL of the sample and incubated in the dark at 37°C for 25 min. The mixtures were then centrifuged at 3,000 × g for 10 min (Thermo Scientific, Waltham, MA, United States). Absorbance was recorded at 593 nm using a FlexStation 3 UV–VIS spectrophotometer (Molecular Devices, Sunnyvale, CA, United States). A Trolox calibration curve (20–250 μM, *R*^2^ = 0.9991) was used, and results were expressed as Trolox equivalents.

##### 2.7.3.3 Cupric ion reducing antioxidant capacity assay

The CUPRAC assay was performed as described by Çelik et al. (2010). A reaction mixture containing 50 μL of CuCl2 (10 mM), 50 μL of neocuproine (7.5 mM), and 50 μL of ammonium acetate buffer (1 M, pH 7.0) was combined with 60 μL of the sample or standard at different concentrations. Following 20 min incubation at room temperature, samples were centrifuged at 3,000 × g for 10 min, and absorbance was measured at 450 nm. A Trolox calibration curve (0.125–2.0 mM, *R*^2^ = 0.9976) was prepared from 2 mM Trolox stock solutions, and results were expressed as mmol Trolox equivalents per gram of dry sample.

##### 2.7.3.4 Trolox equivalent antioxidant capacity assay

The TEAC assay was conducted following the method of Re et al. (1999) with slight modifications. ABTS^+^ radical cation stock solution was generated by reacting 7 mM ABTS with 2.45 mM potassium persulfate and allowing the mixture to stand in the dark at room temperature for 12–16 h. The working ABTS^+^ solution was prepared by diluting the stock with ethanol to achieve an absorbance of ∼0.70 at 734 nm. For the assay, 60 μL of sample or standard was mixed with 540 μL of the ABTS^+^ solution and incubated in the dark for 20 min, followed by centrifugation at 3,000 × g for 10 min. Trolox standards (20–200 μM, *R*^2^ = 0.997) were used to construct a calibration curve, and results were expressed as mmol Trolox equivalents per gram of dry sample.

##### 2.7.3.5 Nitric oxide scavenger

Nitric oxide (NO) generation from sodium nitroprusside (SNP) was assessed following the method reported by Sihag et al. (2022). Briefly, a 1.0 mL reaction mixture containing 5 mM SNP in phosphate-buffered saline (PBS, pH 7.3), with the sample (1 mg/mL), was incubated at 25°C for 2.5 h. A control sample was prepared under the same conditions by replacing the test compounds with an equivalent volume of PBS. After incubation, 0.2 mL of the reaction mixture was combined with 0.2 mL of Griess reagent (comprising 2% sulphanilamide, 5% phosphoric acid, and 0.1% naphthylethylenediamine dihydrochloride). The absorbance of the resulting chromophore, formed through the diazotization of nitrite with sulphanilamide and subsequent coupling with naphthylethylenediamine, was measured at 546 nm. Gallic acid served as the reference antioxidant.

### 2.8 Hemocompatibility evaluation

#### 2.8.1 Hemolysis assay

The hemolytic activity of the samples was evaluated using sheep erythrocytes as previously described Voicu et al. (2025), with minor modifications. Briefly, 9 mL of freshly collected blood was mixed with 1 mL of 10% citric acid dextrose anticoagulant and centrifuged at 5,000 rpm for 10 min. The supernatant was discarded, and the erythrocyte pellet was washed three times with phosphate-buffered saline (PBS; 0.2 M, pH 7.4) before resuspension in sterile saline solution (0.9%). Aliquots of 100 μL of test samples at various concentrations (5, 10, 20, and 50 mg/mL) were added to 400 μL of 10% erythrocyte suspension. The mixtures were incubated for 1 h at 37°C, followed by centrifugation at 5,000 rpm for 10 min. Hemolysis was quantified by measuring the absorbance of the supernatant at 540 nm using a FlexStation 3 UV–VIS spectrophotometer (Molecular Devices, Sunnyvale, CA, United States). Erythrocytes treated with 1% Triton X-100 and PBS (1×) served as positive and negative controls, respectively.

#### 2.8.2 Anti-hemolytic assay

The anti-hemolytic activity of extracellular metabolites was determined according to the spectrophotometric method of Geana et al. (2023) with modifications. Ram blood was collected in acid citrate dextrose (ACD) tubes and centrifuged at 5,000 rpm for 10 min. The erythrocyte pellet was washed three times with PBS (0.2 M, pH 7.4) and resuspended in isotonic saline solution (0.9%). Test samples (50 μL; 5, 10, and 20 mg/mL in PBS, pH 7.4) were mixed with 200 μL of 10% erythrocyte suspension and incubated at 37°C for 20 min. Oxidative hemolysis was induced by adding 100 μL of AAPH (2,2’-azobis(2-amidinopropane) dihydrochloride; final concentration 200 mM) and incubating for 4 h at 37°C. After incubation, the mixtures were centrifuged at 5,000 rpm for 10 min, and the absorbance of the supernatant was recorded at 540 nm. The extent of hemolysis was normalized to the AAPH-treated control (100%), whereas erythrocytes incubated with PBS in place of AAPH served as the negative control. Each experiment was performed in triplicate. Ascorbic acid (5, 10, and 20 mg/mL) was included as a positive antioxidant control. The percentage inhibition of hemolysis was calculated using the following equation: Hemolysis (%) = (A _*sample/AAPH*_ - A _*PBS*_) × 100/(A _*Triton X* –100_ - A _*PBS*_).

### 2.9 Adhesion to Caco-2 cells

Adhesion of Gt28L to Caco-2 cells was evaluated following Wang et al. (2022) with minor modifications. Caco-2 cells (HTB-37, ATCC), an *in vitro* model for differentiated enterocytes, were cultured in RPMI 1640 medium (Thermo Fisher, #11875093) supplemented with 10% FBS and penicillin/streptomycin at 37°C, 5% CO2 for 14–21 days to allow full differentiation. Cells (2 × 105/mL) were seeded in 24-well plates (Fisherbrand, #FB012929) until confluent (∼80–100%). Gt28L cells were inoculated at MOI 10:1 or 100:1 and incubated for 2 h at 37°C. Non-adherent bacteria were removed by PBS washing, and adherent cells were lysed with 0.1% Triton X-100 (in PBS). Released bacteria were serially diluted and plated on MRS agar. Adhesion ability (%) was calculated as (At/Ax) × 100, where At = adherent CFU and Ax = initial CFU (Wang et al., 2022). *E. coli* ATCC11229 was used as a non-probiotic control; adherence > 20% was considered high. For microscopy, Caco-2 cells were cultured on coverslips in 24-well plates and co-incubated with bacteria as described. After 2 h, wells were washed with PBS, fixed with methanol (15 min), air-dried, and stained with Giemsa. Coverslips were dried overnight, and adhesion was visualized using a Zeiss PrimoStar 3 microscope at 100 × magnification.

### 2.10 Statistical analysis

All experiments were performed in triplicate, and data are expressed as mean ± standard deviation (SD). Statistical analyses were conducted using GraphPad Prism version 10 (GraphPad Software, San Diego, CA, United States). Antioxidant activity data were analyzed using an unpaired *t*-test. Comparisons among antioxidant assays (CUPRAC, FRAP, and TEAC) were performed using two-way analysis of variance (ANOVA), followed by Tukey’s *post-hoc* test. Data normality was assessed using the Shapiro–Wilk test and was confirmed for *p* > 0.05. For nitric oxide scavenging assays and hemolytic activity, two-way ANOVA with Geisser–Greenhouse correction was applied, followed by Tukey’s *post-hoc* test. Statistical significance was defined as *p* < 0.05.

## 3 Results and discussion

### 3.1 Genomic features and functional traits of Gt28L

**FIGURE 2:**
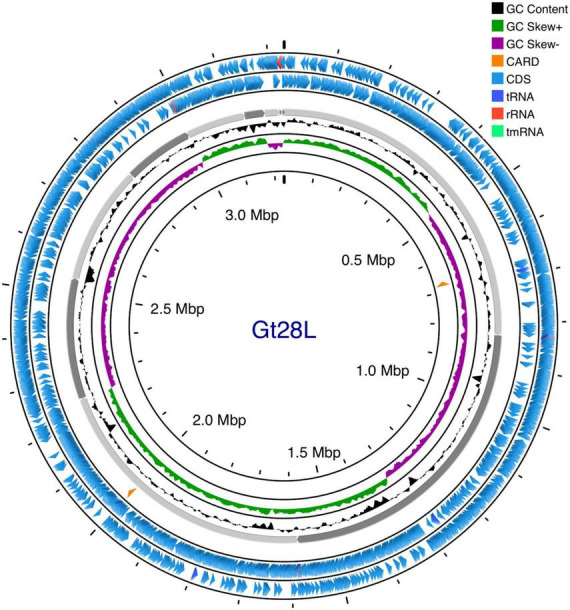
Circular genome diagram of *L. plantarum* Gt28L. Legend starting from the outermost ring: Ring 1, Prokka Annotation (+); Ring 2, Prokka Annotation (-); Ring 3, CARD RGI Results (+); Backbone (Contigs); Ring 5, GC Content; Ring 6, GC Skew; Ring 7, CARD RGI Results (-).

**FIGURE 3:**
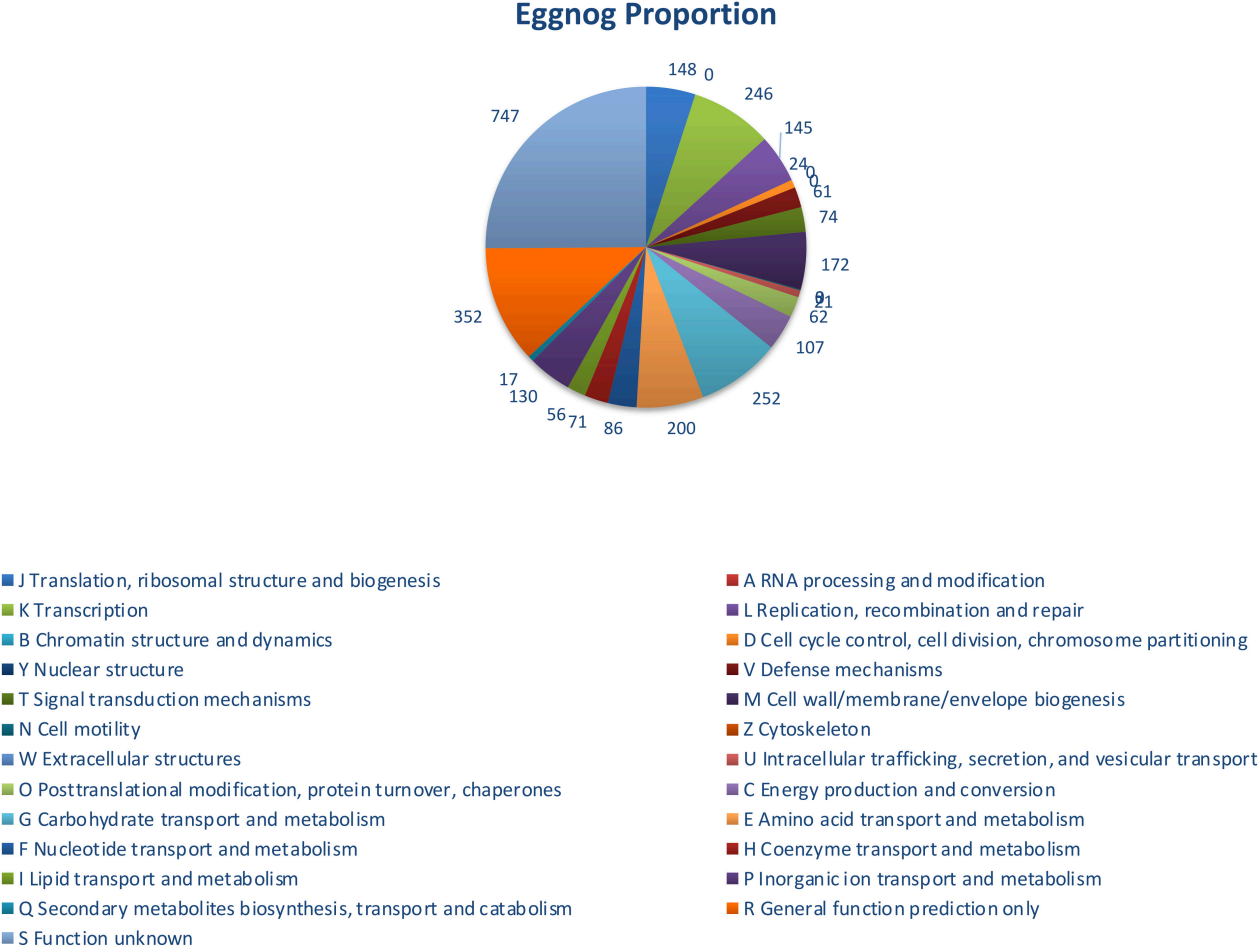
Functional annotation of predicted proteins in strain Gt28L based on EggNOG classification. The pie chart displays the distribution of gene functions across COG (Clusters of Orthologous Groups) categories. The most represented functions include translation, ribosomal structure, and biogenesis, transcription, and amino acid transport and metabolism.

The genome of Gt28L isolated from the Amazonian star apple fruit exhibits a compact and streamlined architecture suggestive of ecological adaptation to a fruit-associated niche. Bioinformatic analysis of the whole-genome sequencing data resulted in a complete genome assembly of 3,231,781 bp, with an average GC content of 44.54%, consistent with the species-specific genomic composition of *L. plantarum* and indicative of a stable genomic architecture (Table 1). The strain harbors 64 tRNA genes, indicating a potentially constrained codon usage adapted to the consistent nutrient landscape of its environment. Essential rRNA and tmRNA genes are conserved, while the absence of CRISPR-Cas arrays, frequently detected in other *L. plantarum* strains, may reflect a reduced phage burden in this niche. Despite lacking CRISPR-Cas, Gt28L retains three intact prophages (PHAGE_Lactob_Lj965, PHAGE_Lactob_phig1e, and PHAGE_Lactob_Sha1), which could enhance its ecological competitiveness, genomic plasticity, and stress resistance (Ventura et al., 2003; Luo et al., 2014; Martínez et al., 2022). Importantly, the genome lacks plasmids, transferable ARGs and known virulence factors, underscoring its genomic stability and biosafety. Taxonomic classification based on BLASTN analysis of the full contig sequence showed 100% identity at the genus level to *Lactobacillus* spp. Average Nucleotide Identity (ANI) analysis revealed 99.35% nucleotide identity and 89.60% alignment coverage with *L. plantarum* strain r-JN-1 (Supplementary Figure 1), supporting species-level assignment. A detailed genome map is shown in Figure 2, and functional annotation statistics across multiple databases are summarized in Table 2. The most abundant functional category is “General function prediction only” (R), comprising 747 genes, reflecting the presence of genes with predicted roles yet lacking experimental characterization, a typical feature of underexplored probiotic genomes (Figure 3). This is followed by “Function unknown” (S, 352 genes), highlighting a substantial reservoir of potentially novel or strain-specific genes warranting further functional validation (Douillard and de Vos, 2014). Among the well-defined categories, genes related to “Translation, ribosomal structure, and biogenesis” (J, 252 genes), “Amino acid transport and metabolism” (E, 200 genes), and “Carbohydrate transport and metabolism” (G, 172 genes) are prominently represented. These functional groups are critical for metabolic adaptability, efficient nutrient acquisition, and survival in competitive environments such as the gastrointestinal tract or fermented matrices. Additional enrichment in “Transcription” (K, 246 genes) and “Posttranslational modification, protein turnover, chaperones” (O, 145 genes) suggests effective regulatory and stress-response mechanisms. Furthermore, the presence of genes linked to “Defense mechanisms” (V, 107 genes) and “Cell wall/membrane/envelope biogenesis” (M, 130 genes) reinforces the strain’s potential for probiotic activity, including resilience to environmental stress and host interaction.

**TABLE 1:** Genome features of *L. plantarum* Gt28L.

Strain	*L. plantarum* Gt28L
Source	*Chrysophyllum cainito* (star apple) fruit
Genome length (bp)	3,231,781
Plasmids	None
GC content (%)	44.54
Total number of genes	3,143
Coding genes	3,079
tRNA number of assembled genome	64
rRNA number of assembled genome	8
tmRNA number of assembled genome	1
CRISPR-Cas array*	0
Prophage (intact region)**	3
Antibiotic acquired genes***	None
Pathogenicity****	Non-human pathogen

*CRISPRFinder (https://crisprcas.i2bc.paris-saclay.fr/CrisprCasFinder/Index);

**PHAge Search Tool Enhanced Release (PHASTER) (https://phaster.ca);

***ResFinder 4.1 (https://genepi.food.dtu.dk/resfinder);

****PathogenFinder. (https://genepi.food.dtu.dk/pathogenfinder).

**TABLE 2:** Gene annotation summary.

Num of genes (#)	CARD	MetaCyc	PHI	CAZy	VFDB	SwissProt	KEGG	COG
3078	49(1.59)	494(16.05)	254(8.25)	3,078(100.00)	96(3.12)	1,192(38.73)	3,078(100.00)	2,232(72.51)

CARD, Comprehensive Antibiotic Resistance Database; MetaCyc, database that contains pathways involved in both primary and secondary metabolism; PHI, The Pathogen-Host Interaction database is a biological database that contains curated information on genes experimentally proven to affect the outcome of pathogen-host interactions; CAZy, Carbohydrate-active enzyme; VFDB, virulence factor database; SwissProt; KEGG, Kyoto Encyclopedia of Genes and Genomes; COG, Clusters of Orthologous Groups of proteins.

### 3.2 Safety evaluation based on WGS analysis

The distribution of antimicrobial resistance (AMR) features across three categories, ARG families, resistance mechanisms, and drug classes is illustrated in Supplementary Figure S2. The analysis indicates that the detected resistance traits are predominantly associated with intrinsic, chromosomally encoded mechanisms, which are commonly observed in lactic acid bacteria and are not linked to horizontal gene transfer. Specifically, antibiotic target alteration and antibiotic efflux represent the most frequently identified resistance mechanisms. At the ARG family level, glycopeptide resistance–associated gene clusters and ATP-binding cassette (ABC) efflux systems were the most abundant. These findings reflect the expected intrinsic resistance profile of the strain, rather than the presence of clinically relevant or transferable resistance determinants. The comparative resistome analysis of the Gt28L strain identified several ARGs, including those related to ribosomal protection (*fusA*, *rpoB*) and translation elongation (*EFTu*), with prevalence values reaching over 70% (Supplementary Table 5). The presence of vancomycin-resistance associated genes such as *vanY* and *vanT*, typically chromosomally encoded and intrinsic in *L. plantarum*, should not raise safety concerns, as these ARGs are non-transferable and part of the species’ natural resistome (Ammor et al., 2007). *van*Y was confirmed by RGI analysis with 95.90% sequence identity. Importantly, efflux-related genes such as *qacJ*, *mdtG*, and *poxtA*, which may play roles in resistance to biocides and antimicrobial agents, were also detected. These features suggest environmental adaptability and intrinsic resilience but do not imply pathogenic potential (Hu et al., 2013; Ojha et al., 2023). While *L. plantarum* is generally considered a safe microorganism with low AMR risk (Douillard and de Vos, 2014), the detection of multiple resistance markers at significant prevalence levels emphasizes the need for thorough genomic screening prior to industrial or clinical use. In addition, similar with other *Lactiplantibacillus* species (Li and Bi, 2025), *tet*O and *tet*A genes were annotated with EggNOG. Moreover, PathogenFinder analysis predicted Gt28L as a non-human pathogen with a 99.95% probability, indicating a highly confident classification.

Furthermore, to assess safety, the genome of Gt28L was screened against the VFDB (Supplementary Table 6). Although several sequences returned VFDB annotations, these hits corresponded only to low-identity matches (40–76.8%) involving housekeeping, metabolic, stress-response, or cell-surface biosynthesis genes commonly found in *L. plantarum* and other GRAS/QPS lactic acid bacteria (Colautti et al., 2022). None of the annotations represented bona fide virulence determinants such as toxins, hemolysins, cytolysins, invasins, or pathogenicity-associated secretion systems. The genes including Clp proteases, chaperones (DnaK/GroEL), glycolytic enzymes, ABC transporters, and exopolysaccharide biosynthesis genes are essential for bacterial physiology and are well-established features of safe, commensal LAB (Colautti et al., 2022). Likewise, domain-level similarity to *pavA*- or *lap*-like adhesion proteins reflects beneficial mucosal adherence functions, not virulence. Apparent matches to Dot/Icm-like secretion proteins do not imply pathogenic potential, as Gt28L lacks the structural modules required to assemble a functional virulence-associated T4SS.

Phenotypic assays further confirmed the safety of Gt28L the strain was non-hemolytic and non-cytotoxic to epithelial cells. The presence of such homologous genes does not demonstrate virulence, as sequence similarity alone cannot determine their functional role or pathogenic potential (Altavas et al., 2024). Together, these results indicate that the VFDB hits do not correspond to true virulence factors and affirm the overall safety of the Gt28L strain.

Comparative analysis with reference strains *L. plantarum* WCFS1 and *L. plantarum* ST-III revealed a shared core of such genes, suggesting these elements are part of the intrinsic genomic repertoire of the species (Wang et al., 2011). Notably, these genes are involved in key probiotic functions such as epithelial adhesion, stress resistance, and carbohydrate metabolism, supporting their functional conservation for host adaptation rather than pathogenicity. Similar findings have been reported in other *Lactobacillus* strains, reinforcing the concept that the presence of these genes alone does not imply virulence but reflects ecological fitness and commensal lifestyle (Petrova et al., 2016).

### 3.3 Phylogenetic relationship

The phylogenetic analysis places Gt28L within a monophyletic clade comprising other well-characterized *L. plantarum* strains, including strains historically designated as *L. arizonensis* and *L. argentoratensis*. Recent taxonomic studies have reclassified these species as heterotypic synonyms of *L. plantarum* (Zheng et al., 2020), confirming that Gt28L is consistent with *the L. plantarum* species complex (Supplementary Figure 3). The tree topology, supported by high bootstrap values, indicates that Gt28L shares a recent common ancestor with strains such as GCM100194569 and GCM100103221, reflecting conserved genomic architecture within this group. Heatmap metadata further support its genomic stability, with Gt28L exhibiting a genome size (∼3.2 Mb) and GC content (∼44.5%) characteristic of *L. plantarum*, and low standard deviation values indicating minimal structural variability. These parameters reinforce the notion that Gt28L maintains evolutionary coherence with commensal *L. plantarum* species, while also exhibiting a streamlined genome potentially shaped by niche-specific pressures in the Amazonian fruit environment. Such genomic conservation, combined with its distinct ecological origin, positions Gt28L as a valuable reference for studying functional diversification within the *L. plantarum* lineage.

### 3.4 Bacteriocin and secondary metabolite gene clusters in Gt28L

**FIGURE 4:**
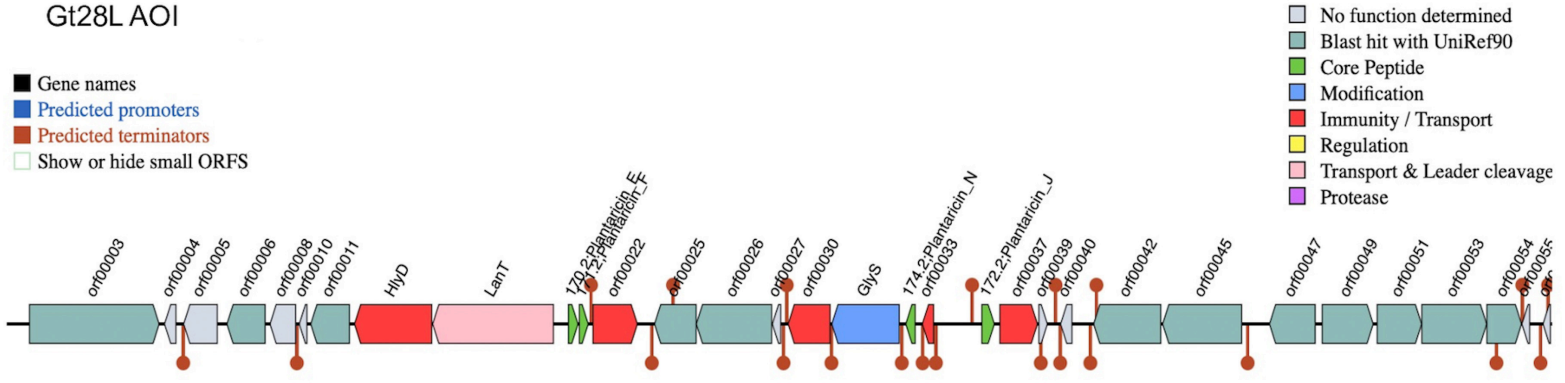
Biosynthetic gene cluster (BGC) organization of Area of Interest (AOI) in strain Gt28L. The figure shows the arrangement of open reading frames (ORFs) within the cluster, with color-coded annotations indicating predicted functions: core peptides (green), modification enzymes (blue), immunity/transport genes (red), regulatory elements (yellow), and transport/leader cleavage components (pink). Promoters (blue bars) and terminators (red bars) are also predicted.

Genome mining revealed a well-defined BGC encompassing multiple genes involved in the synthesis, modification, transport, and immunity of class IIb bacteriocins (Figure 4). The cluster spans approximately 55 open reading frames (ORFs), including several putative bacteriocins, notably *plantaricin_E*, *plantaricin_F*, *plantaricin_N*, and *plantaricin_J* (orf00022, orf00031, orf00033, and orf00037, respectively). These core peptides are characteristic of the two-peptide bacteriocin system previously described in *L. plantarum* C11 and WCFS1, where synergistic action between peptides is required for antimicrobial activity (Anderssen et al., 1998; Heeney et al., 2019). Notably, the presence of multiple predicted core peptides within a single cluster indicates a potentially broad antimicrobial spectrum or multi-targeted mechanism. Associated with these structural genes are dedicated transport and immunity proteins, including *LanT* and *HlyD*, which are typically involved in the secretion and protection mechanisms of bacteriocin-producing strains (Nissen-Meyer et al., 2010). The presence of a glycosyltransferase gene (*GlyS*, orf00030) suggests potential post-translational modification, which may enhance bacteriocin stability or activity, as previously noted in glycosylated bacteriocins of other LAB strains (Todorov, 2009). Compared to other *L. plantarum* strains, the Gt28L cluster displays a higher degree of gene multiplicity and operon organization. For example, in *L. plantarum* WCFS1, the plantaricin locus contains fewer structural genes and is regulated by a well-characterized quorum-sensing system (Heeney et al., 2019), which appears absent or divergent in Gt28L. Moreover, the inclusion of two sets of *plantaricin_E/F*-like peptides in Gt28L suggests a possible gene duplication or horizontal acquisition event, offering a broader antimicrobial capacity. In terms of genomic arrangement, the Gt28L cluster shares synteny with the *pln* loci of several *L. plantarum* strains (Gizachew and Engidawork, 2024), but includes unique ORFs with no currently assigned function, indicating possible strain-specific adaptations or novel components. The abundance of predicted transcriptional terminators and promoters further supports the existence of multiple transcriptional units, suggesting tight regulation and potential responsiveness to environmental stimuli.

In addition, several a diverse secondary metabolite were predicted with antiSMASH (Table 3). A complete list of predicted compounds, category, location, and similarity scores are shown in Supplementary Table 7. Notably, contig 1.1 showed weak to moderate sequence similarity (21-32%) to known polyketide and non-ribosomal peptide clusters, including those associated with falcarindiol, chrysoxanthones, viguiepinol, and bacilysin, which are typically found in *Streptomyces*, *Penicillium*, and *Bacillus* species. Although these clusters displayed low similarity percentages, they may represent cryptic or evolutionarily conserved fragments, possibly involved in the biosynthesis of structurally similar metabolites with antimicrobial potential (Wang et al., 2011). In contrast, contigs 2.1 and 2.2 revealed multiple RiPP-like BGCs showing 16–22% similarity to characterized bacteriocins such as coagulin, microcin M and E492, ubericin K, and sublancin 168. Sublancin 168 was previously predicted in the genome of *L. plantarum* UTNG21A but was absent in the reference strain *L. plantarum* WCFS1, reinforcing the hypothesis that strain-specific biosynthetic capabilities, including the production of novel antimicrobial compounds, may be shaped by selective pressures within their native ecological niches (Tenea and Ascanta, 2022). Additionally, contig 7.1 contained a terpene biosynthetic cluster with high similarity (up to 45%) to carotenoid-producing pathways from *Rhodobacter* and *Streptomyces*, indicating potential antioxidant or stress adaptation roles. Taken together, these genomic features highlight the capacity of Gt28L to produce a diverse array of secondary metabolites, including multiple bacteriocin-like peptides and terpenes. Importantly, we recently demonstrated the antimicrobial activity of Gt28L *in vitro* against the multidrug-resistant *E. coli* L1PEag1 strain, which validates and supports the *in silico* predictions derived from BGC analysis (Tenea et al., 2025). These findings reinforce the strain potential as a source of novel antimicrobial compounds and its relevance for probiotic and biocontrol applications.

**TABLE 3:** Summary of the secondary metabolites predicted by antiSMASH and biological role.

Contig	Key feature	Biological role	Confidence
1.1	Broad-spectrum hits to polyketides, NRPs, and others	Likely cryptic or ancestral clusters	Moderate
2.1/2.2	RiPPs: microcins, enterocins, bacteriocins	Potential antimicrobial peptides	High
7.1	Carotenoid-terpene cluster	Antioxidant activity, stress adaptation	High

### 3.5 Pangenome analysis

**FIGURE 5:**
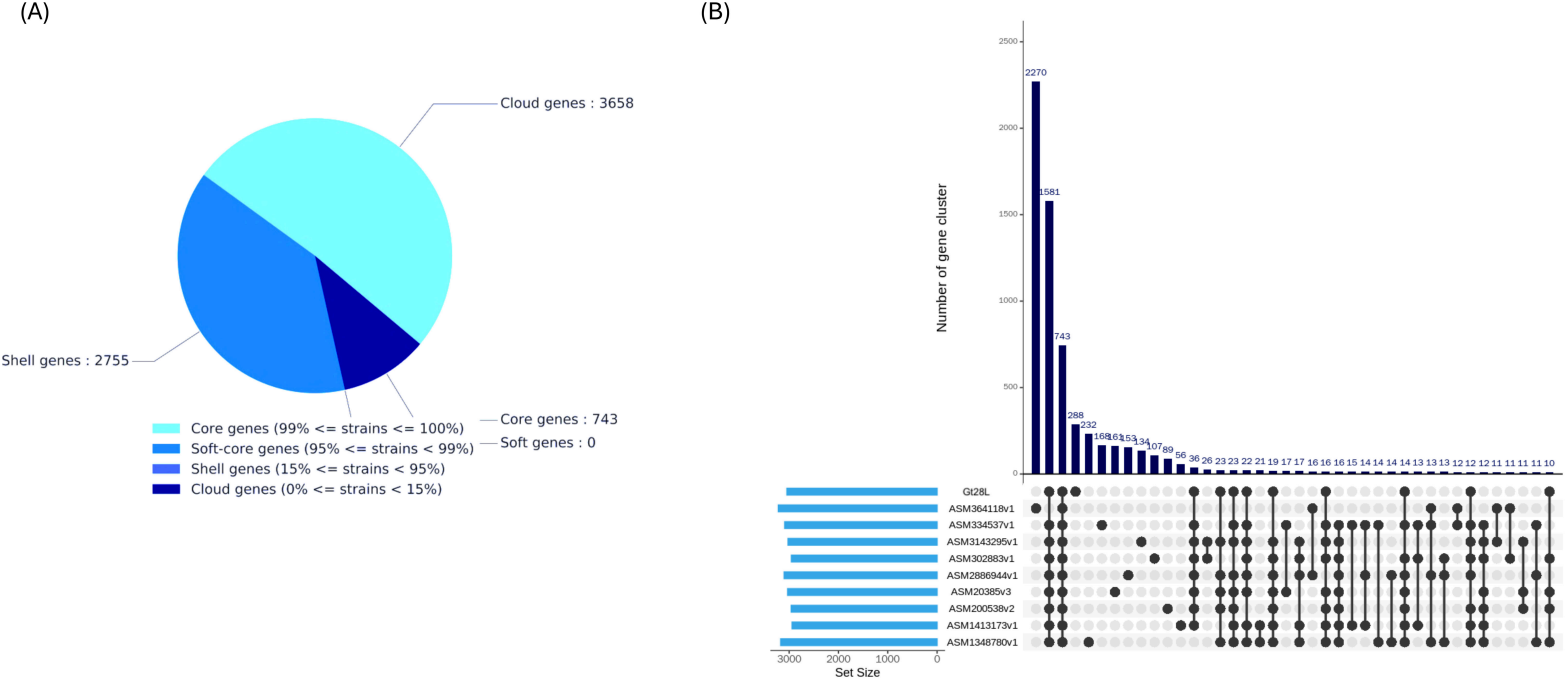
**(A)** Pie chart illustrating the distribution of genes within the Gt28L genome across the pangenome categories: core, soft core, shell, and cloud. These classifications reflect gene conservation levels among compared genomes. **(B)** UpSet plot depicting the presence and overlap of gene clusters among various *Lactobacillus* strains. The upper histogram indicates the number of gene clusters shared across specific strain combinations, while the left-side bars show the total gene cluster count per strain.

The pangenome analysis of *L. plantarum* strains revealed a highly diverse genomic structure, indicative of substantial adaptive potential. Among the total gene pool, only 743 genes (∼14%) were identified as core genes shared by 99–100% of strains (Figure 5A), reflecting the minimal set of conserved functions essential for survival. A larger fraction comprised shell genes (2,755 genes, ∼52%), present in 15–95% of strains, likely conferring niche-specific advantages such as carbohydrate metabolism, stress response, or bacteriocin synthesis. Cloud genes accounted for 3,658 genes (∼34%), representing highly variable, strain-specific elements often associated with horizontal gene transfer, prophages, or mobile genetic elements. The absence of soft-core genes underscores the pronounced dichotomy in gene conservation and the overall flexibility of the *L. plantarum* genome, consistent with its open pangenome and broad ecological range across fermented foods, plant surfaces, and animal gastrointestinal tracts (Choi et al., 2021; Carpi et al., 2022). The UpSet plot shows that most gene clusters (2,270) belong to the core genome, and Gt28L does not dominate any large unique cluster sets (Figure 5B). Roary matrix analysis and the phylogenetic tree further demonstrate Gt28L divergence, with a distinctive pattern of accessory gene presence and absence (Supplementary Figure 4), suggesting potential ecological or functional specialization. The Gt28L genomic architecture reflects adaptation to its fruit-associated niche and likely underpins its unique probiotic traits compared to other *L. plantarum* strains. Its compact, streamlined genome, combined with an abundant accessory gene pool, facilitates rapid adaptation to variable nutrient and stress conditions. Enrichment in genes related to amino acid transport and metabolism, carbohydrate utilization, and stress-response pathways supports environmental resilience and competitive colonization, while the absence of plasmids, transferable antibiotic resistance genes, and canonical virulence factors ensures biosafety. Extensive bacteriocin and RiPP-like clusters, alongside secondary metabolite pathways such as terpene and carotenoid biosynthesis, may provide broad antimicrobial and antioxidant capabilities, enhancing survival in microbially diverse niches. Compared to reference strains like WCFS1 or ST-III, often optimized for gut or dairy environments, Gt28L exhibits a strain-specific integration of accessory genes that support both ecological versatility and metabolically driven probiotic functions, including adhesion and stress tolerance. Thus, these genomic features highlight how Gt28L distinctiveness translates into functional traits enabling adaptation to its native niche while promoting health-supportive and antimicrobial properties.

### 3.6 Intracellular metabolites profile and functional traits of Gt28L

#### 3.6.1 Distinctive metabolites support probiotic potential

The metabolic capacity of *L. plantarum* strains is highly strain-dependent, shaped by genomic composition, substrate availability, and environmental conditions (Pulido-Mateos et al., 2024). The metabolomic profile of Gt28L revealed a broad biosynthetic repertoire, including amino acids such as valine, leucine, isoleucine, alanine, proline, glutamic acid, γ-aminobutyric acid (GABA), and lactamide (Table 4). These metabolites are functionally relevant for gut health, contributing to mucosal integrity, immune modulation, and inter-microbial signaling (Chatsirisakul et al., 2025; Zhou et al., 2025). Notably, lactamide (2-hydroxypropanamide) was uniquely detected in Gt28L and not in the MRS control, suggesting a strain-specific metabolic transformation possibly derived from lactic acid or related fermentation intermediates. This feature points to unique enzymatic or regulatory mechanisms potentially linked to the probiotic functionality of Gt28L. Genomic analysis confirmed the presence of the glutamate decarboxylase (GAD) gene cluster, supporting the biosynthesis of GABA from L-glutamate. While this pathway is broadly distributed among *L. plantarum* strains, quantitative differences in production are common (Abdel-motaal et al., 2024). GABA is of particular interest due to its multifunctional health-promoting roles, including stress relief, blood pressure regulation, antioxidant activity, and possible anti-diabetic effects (Kim et al., 2022; Iorizzo et al., 2023; Yao et al., 2025; Barakat and Aljutaily, 2025). The functional annotation of Gt28L suggests it may serve as a promising candidate for GABA-enriched postbiotic development, though targeted validation is needed.

**TABLE 4:** List of compounds and class detected by GC/MS-QTOF in the Gt28L Met-Int.

Compound	Retention time (min)	Formula	Area	
			Gt28L	MRS medium
**Alcohols and polyols**				
Myo-inositol	19.109	C6H12O6	166519	0
**Amino acids and derivatives**				
Valine	7.357	C5HNO2	996389	464286
Alanine	7.630	C3H7NO2	749026	0
Lactamide	8.231	C3H7NO2	535178	0
Leucine	8.385	C6H13NO2	1579974	846376
Isoleucine	8.705	C6H13NO2	1269819	397654
Serine	9.893	C3H7NO3	332191	387427
Threonine	10.453	C4H9NO3	0	671932
Proline	10.499	C5H9NO2	278131	0
Glycine	10.634	C2H5NO2	666073	0
Aspartic acid	12.144	C4H7NO4	230837	401118
Pyroglutamic acid	13.443	C5H7NO3	2807458	4850814
Glutamic acid	13.484	C5H9NO4	794659	0
Aminobutanoic acid	13.525	C4H9NO2	412021	0
Phenylalanine	13.714	C9H11NO2	339455	436776
**Benzenoids**				
Benzoic acid	9.733	C7H6O2	3340	8374
**Carbohydrates and carbohydrate conjugates**				
Glyceric acid	10.976	C3H6O4	80006	239108
Ribose	15.240	C5H10O5	0	22114
Fructose	17.550	C6H12O6	23086143	64637933
Glucose	17.825	C6H12O6	119587083	229094856
Galactitol	18.158	C6H14O6	150316	0
Maltose	24.792	C12H22O11	34362538	4058607
**Fatty acyl**				
2-Hydroxybutyric acid	8.008	C4H8O3	5944	28187
3-Hydroxybutyric acid	8.476	C4H8O3	0	42103
Hydroxy-3-Methylbutyric Acid	8.576	C5H10O3	59900	16269
Hydroxyisocaproic Acid	9.598	C6H12O3	218829	0
2-hydroxy-3-methylvaleric acid	9.705	C6H12O3	135577	0
Palmitic acid	18.928	C16H32O2	92228	0
Stearic acid	20.715	C18H36O2	263516	0
**Hydroxy acids and derivatives**				
Lactic acid	6.988	C3H6O3	111607839	23767779
Glycolic acid	7.201	C2H4O3	164924	217812
3-hydroxypropanoic acid	8.235	C3H6O3	0	267959
Dihydroxy-2-methylpropanoic acid	10.842	C4H8O4	136302	315811
2,4-dihydroxybutanoic acid	12.056	C4H8O4	139708	370426
3,4-dihydroxybutanoic acid	12.300	C4H8O4	39787	119922
Malic acid	13.027	C4H6O5	190031	141781
**Lactones**				
3-deoxy-ribo-hexonic acid lactone	16.404	C6H10O5	117399	0
**Non-metal phosphates**				
Phosphate	10.256	H3O4P	0	29743
**Organic acids and derivatives**				
Pyruvic acid	6.811	C3H4O3	109958	24145
Malonic acid	9.134	C3H4O4	0	30971
Succinic acid	10.655	C4H6O4	712274	1246951
Glycerol-3-phosphate	16.292	C3H9O6P	798521	0
**Phenylpropanoic acids**				
Phenyllactic acid	14.212	C9H10O3	516546	107590
3-(4-hydroxyphenyl)lactic acid	17.679	C9H10O4	1012472	0
**Pyridines and derivatives**				
Nicotinamide	12.833	C6H6N2O	55242	0

Area refers to the integrated signal of a given peak in the chromatogram.

Beyond amino acid metabolism, Gt28L synthesizes bioactive phenolic acids such as phenyllactic acid (PLA) and 3-(4-hydroxyphenyl)lactic acid, well-established antimicrobial metabolites that enhance competitiveness in the gut ecosystem (Pulido-Mateos et al., 2024). The production of lactic acid further contributes to pathogen inhibition through pH reduction, a hallmark of probiotic function (Stavropoulou and Bezirtzoglou, 2020). Moreover, the detection of carbohydrates (fructose, glucose, maltose, galactitol) metabolic pathways highlights the strain’s fermentative adaptability, which may support biofilm formation, stress resilience, and persistence within the gastrointestinal environment (Sharma et al., 2023). Fatty acids such as palmitic and stearic acid, along with hydroxy-fatty acids, indicate active lipid metabolism that can influence membrane fluidity and antimicrobial activity (O’Shea et al., 2012). The additional capacity to synthesize nicotinamide (vitamin B3) further underscores its roles in maintaining the redox balance and in immunomodulation (Doroftei et al., 2020).

**FIGURE 6:**
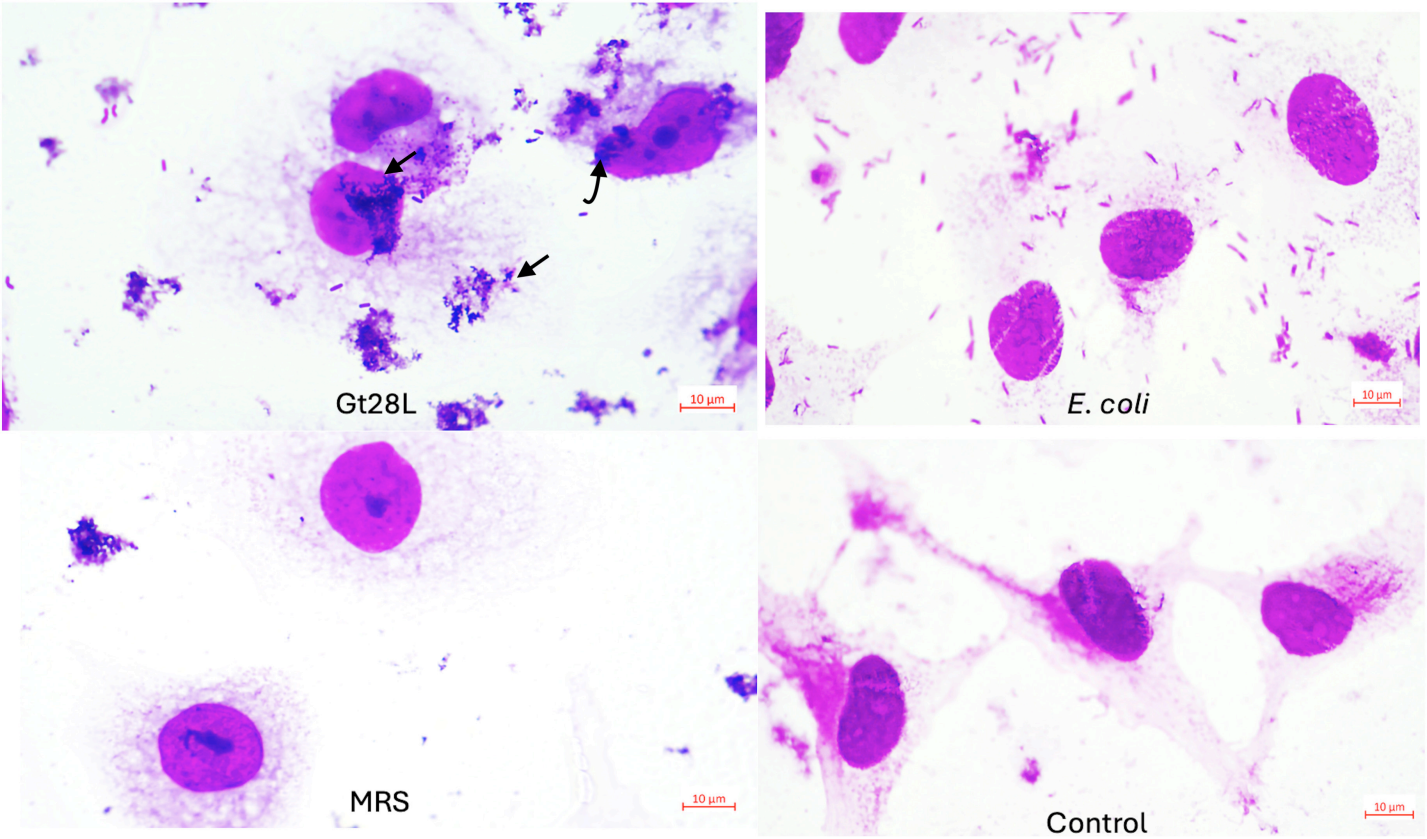
Adherence to Caco-2 cells. Representative microscopic observation (100×).

Importantly, functional traits must be paired with effective host interaction to qualify a strain as probiotic. In this regard, Gt28L demonstrated enhanced colonization potential, indicated by a strong adhesion to Caco-2 cells, 1.68-fold higher than that observed for the non-probiotic control *E. coli* ATCC11229. Microscopic observations supported these findings, showing localized and aggregative adherence patterns supporting for advantageous colonization traits (Wang et al., 2022), in contrast to the diffuse on, recorded for *E. coli* (Figure 6).

#### 3.6.2 Metabolic versatility and amino acid pathway enrichment in Gt28L

The KEGG pathway enrichment network of *L. plantarum* Gt28L reveals a distinctive metabolic profile characterized by a strong enrichment in amino acid metabolism, particularly in pathways related to the degradation and biosynthesis of valine, leucine, isoleucine, glycine, serine, and threonine (Supplementary Figure 5). Compared to other well-characterized *L. plantarum* strains such as WCFS1 and ST-III, which predominantly emphasize carbohydrate metabolism and stress-related pathways due to their gut or dairy origins (Kleerebezem et al., 2003; Wang et al., 2011), Gt28L exhibits a more interconnected network centered around energy-efficient pathways like glyoxylate and dicarboxylate metabolism and pyruvate metabolism. The enrichment in pantothenate and CoA biosynthesis, glutathione metabolism, and methane/butanoate pathways suggests enhanced capabilities for oxidative stress response and redox homeostasis, potentially reflective of ecological pressures encountered on fruit surfaces. This configuration implies that Gt28L has adapted its metabolic network for environmental flexibility and survival in nutrient-variable, oxidative microenvironments typical of plant-associated niches. The top 25 pathways were enriched with functional metabolites at a statistically significant level (*p* < 0.05), as determined by Metabolite Set Enrichment Analysis (MSEA), which identified biologically coherent trends within the metabolomic dataset (Supplementary Figure 6). These enriched pathways underscore Gt28L substantial capacity for nitrogen assimilation, amino acid interconversion, and survival under nutrient-limited conditions, as well as its potential role in host metabolic interactions in symbiotic niches. In comparison to other *Lactobacillus* species, Gt28L exhibits a unique and extensive engagement in branched-chain amino acid (BCAA) metabolism, encompassing both biosynthesis and degradation of valine, leucine, and isoleucine, as well as glycine-serine-threonine pathways, indicating its pivotal role in amino acid turnover and possible modulation of host protein metabolism (Pulido-Mateos et al., 2022). Although BCAA activity is a hallmark of lactobacilli, the simultaneous enrichment of glyoxylate and dicarboxylate metabolism, pyruvate metabolism, and the TCA cycle in Gt28L suggests a higher degree of metabolic plasticity than that observed in fermentative specialists like *L. delbrueckii* or *L. bulgaricus* (Rajanikar et al., 2021). Moreover, secondary metabolite biosynthesis pathways pf Gt28L distinguish it from most *Lactobacillus* strains (Hernández Figueroa et al., 2024). Unlike *L. rhamnosus* and *L. casei*, which are predominantly geared toward carbohydrate fermentation and lactate production, Gt28L shows broad integration across amino acid, nucleotide, and energy metabolism pathways, indicating enhanced ecological adaptability and probiotic potential.

#### 3.6.3 Metabolites of therapeutic importance

Genomic and metabolic profiling of Gt28L revealed its ability to synthesize several bioactive metabolites, including benzoic acid, PLA, and 3-(4-hydroxyphenyl) lactic acid (HPLA) (Table 4). These compounds, often derived from plant phenolics or amino acid metabolism, contribute significantly to the antimicrobial, antioxidant, and therapeutic potential of specific strains. Benzoic acid can be produced by some *L. plantarum* strains through the degradation of plant-derived phenolic compounds, such as cinnamic acid derivatives commonly found in fermented vegetables and fruits. Its antimicrobial effect is well established, particularly under acidic conditions, where it disrupts microbial pH homeostasis by entering cells in its undissociated form and acidifying the cytoplasm (Barasker et al., 2024). This makes benzoic acid-producing strains valuable in food biopreservation, enhancing the safety and shelf-life of fermented products. Beyond its antimicrobial capacity, benzoic acid has shown anti-inflammatory effects, including therapeutic potential in reducing inflammation associated with intestinal infections such as campylobacteriosis in animal models (Du et al., 2024). Although not directly contributing to gut colonization, the anti-inflammatory properties of benzoic acid can aid in managing gut health, particularly under inflammatory conditions (Anantharaju et al., 2017).

PLA, a product of phenylalanine metabolism, is commonly produced by *L. plantarum* strains, especially when phenylalanine or its precursor phenylpyruvic acid is available in the medium (Shakya et al., 2022). PLA displays broad-spectrum antimicrobial and antifungal activity against both Gram-positive and Gram-negative bacteria, including *Listeria monocytogenes*, *Salmonella enterica*, and *E. coli* O157:H7, as well as fungi like *Candida*, *Fusarium*, and *Aspergillus* (Crossley et al., 2024). Its mechanisms involve membrane damage and DNA intercalation. PLA also inhibits biofilm formation a critical trait in mitigating persistent infections in both food and host environments (Shakya et al., 2022). Additionally, PLA has been reported to possess anti-inflammatory and immunomodulatory properties, suggesting a role beyond antimicrobial activity in host interaction and immune regulation (Crossley et al., 2024). The ability of *L. plantarum* strains to produce PLA thus enhances their value both as food biopreservatives and as contributors to pathogen control and immune balance in the gut.

The presence of HPLA, a key intermediate derived from tyrosine metabolism, was confirmed by GC-MS (Table 4). Although HPLA is generally considered less effective than PLA in terms of direct antimicrobial activity (Valerio et al., 2004), it exhibits a distinct set of biofunctional properties that enhance its relevance in host-microbe and metabolic interactions. HPLA has been shown to possess strong antioxidant capacity, effectively neutralizing reactive oxygen species (ROS) and mitigating oxidative stress at the cellular level (Zhang et al., 2025). This antioxidant function is particularly important in the context of gut health, where oxidative imbalance can disrupt epithelial integrity and microbial homeostasis. In addition to its antioxidative potential, HPLA also exerts anti-inflammatory effects and has demonstrated hepatoprotective properties in preclinical models. Recent studies indicate that HPLA can alleviate alcohol-induced liver injury by reducing hepatic oxidative damage and suppressing lipid accumulation, mechanisms that are central to its proposed role in gut-liver axis modulation (Zhang et al., 2025). These findings suggest that beyond antimicrobial defense, Gt28L-derived metabolites such as HPLA may contribute to systemic host benefits through redox regulation, metabolic protection, and anti-inflammatory signaling. Thus, the production of HPLA by Gt28L adds functional depth to its probiotic profile and supports its potential application in managing oxidative stress–related gastrointestinal and hepatic disorders. Thus, these metabolites highlight the multifunctional roles of Gt28L strain. The presence of these metabolites in specific strains underscores the importance of metabolic profiling in selecting probiotic candidates for therapeutic or food-related applications.

The genomic and metabolomic data collectively reveal that Gt28L functional repertoire is tightly coupled to its unique biosynthetic potential and ecological adaptation. The presence of a well-defined class IIb bacteriocin cluster, including multiple plantaricin-like peptides (PlnE, PlnF, PlnN, PlnJ) and associated transport and immunity proteins (LanT, HlyD), directly correlates with the strain’s demonstrated antimicrobial activity against multidrug-resistant *E. coli* L1PEag1 (Tenea et al., 2025). The coexistence of duplicated PlnE/F-like genes and RiPP-like clusters suggests synergistic or additive effects, potentially broadening the antimicrobial spectrum. Complementing this, metabolomic analysis revealed abundant production of phenolic acids such as phenyllactic acid (PLA) and 3-(4-hydroxyphenyl)lactic acid (HPLA), compounds known to inhibit Gram-positive and Gram-negative pathogens and biofilm formation. Mechanistically, these metabolites are linked to amino acid catabolism pathways (phenylalanine and tyrosine), whose genomic signatures are enriched in Gt28L, indicating that accessory genes encoding amino acid transporters, enzymes, and regulatory elements are functionally expressed. Similarly, the detection of GABA and lactamide corresponds with the presence of the glutamate decarboxylase (GAD) gene cluster and putative enzymes involved in lactic acid derivatives biosynthesis, providing a genomic basis for strain-specific metabolic transformations. Secondary metabolite BGCs for terpenes and carotenoids may underpin the observed antioxidant activities of HPLA and other metabolites, while fatty acid and nicotinamide biosynthetic pathways reinforce redox homeostasis and membrane stability. Collectively, these genomic–metabolomic correlations illustrate a mechanistic framework in which Gt28L specialized BGCs and accessory genes translate into the production of bioactive compounds that confer antimicrobial, antioxidant, and host-beneficial properties, highlighting a direct link between genome architecture, metabolite synthesis, and probiotic functionality.

#### 3.6.4 ADME properties, toxicity, and pharmacological activity of Gt28L-derived metabolites

The *in silico* ADME profiling of metabolites produced by Gt28L suggests generally favorable pharmacokinetic properties, providing preliminary insights into their potential bioavailability and safety. Several low-molecular-weight amino acids (e.g., valine, alanine, leucine) and organic acids (e.g., lactic, pyruvic, and succinic acids) were predicted to exhibit high gastrointestinal (GI) absorption, indicating potential oral availability under physiological conditions (Supplementary Table 8). In contrast, larger saccharides (e.g., maltose, glucose, fructose) and highly polar compounds such as myo-inositol showed low predicted GI absorption, likely reflecting molecular size and polarity constraints inherent to passive diffusion models. Predicted blood–brain barrier (BBB) permeability was limited for most metabolites, with exceptions including benzoic, palmitic, and stearic acids, suggesting minimal central nervous system (CNS) exposure for many compounds. A small subset of metabolites, primarily fatty acids, were predicted to act as P-glycoprotein (P-gp) substrates, indicating a potential for efflux-related modulation of bioavailability (Kadry et al., 2020). Most metabolites complied with Lipinski’s Rule of Five, commonly used as an indicator of drug-likeness, with deviations largely confined to compounds with higher molecular weight or increased lipophilicity (Choy and Prausnitz, 2011). Similar trends have been reported for metabolites identified in *Streptococcus thermophilus*, supporting the broader applicability of these predictive frameworks across lactic acid bacteria–derived metabolites (Sudheer et al., 2025). Gt28L-derived metabolites displayed generally favorable bioavailability scores (∼0.55 for smaller molecules), whereas larger or highly polar metabolites scored lower (∼0.17). Synthetic accessibility values ranged from 2.2 to 3.3, indicating moderate synthetic tractability. These attributes collectively reinforce the therapeutic potential of Gt28L metabolites and are consistent with findings that highlight strain-specific metabolic contributions to probiotic function and application (Iarusso et al., 2025).

Toxicological *in silico* assessment further supports the safety of Gt28L metabolites. Most compounds exhibit high oral LD50 values (> 2,000 mg/kg in rats), placing them within GHS Categories 4 or 5, indicative of low acute toxicity (Supplementary Table 9). None of the major metabolites were associated with hepatotoxic, carcinogenic, immunotoxic, mutagenic, or cytotoxic risks, corroborating their safety for human use. Common dietary metabolites such as glucose, fructose, and glutamic acid showed extremely high LD50 values (> 10,000 mg/kg), confirming negligible toxicity under normal consumption levels. Mild irritation potential was noted for compounds like benzoic and glycolic acids at high concentrations, while palmitic acid may have metabolic implications, including lipid dysregulation and obesity risk (Saraswathi et al., 2020). However, the absence of mutagenicity or carcinogenicity among all analyzed metabolites strengthens their safety profile for probiotic and nutraceutical development. These results align with prior studies affirming the safety of *L. plantarum*-derived metabolites in functional food and therapeutic contexts (Wang et al., 2021), with only a few compounds necessitating dose-dependent considerations in clinical formulations. The cytotoxicity assessment of CFS containing postbiotic metabolites from Gt28L confirmed excellent biocompatibility with human colon epithelial cells, as demonstrated by the 3-(4,5-dimethylthiazol-2-yl)-2,5-diphenyltetrazolium bromide assay (MTT) and the lactate dehydrogenase release assay (LDH)(unpublished data).

Predicted pharmacological activities of selected metabolites were further explored using the PASS web server (Supplementary Table 10), which estimates biological activity probabilities based on structural similarity to known compounds. While a Pa > 0.5 suggests a higher likelihood of activity, these outputs represent probabilistic predictions rather than empirical evidence (Sudheer et al., 2025). Phenyllactic acid (PLA), for example, demonstrated predicted multi-target activity, including potential roles in enzyme inhibition, metabolic regulation, and anti-inflammatory pathways, consistent with prior experimental reports describing its antimicrobial and immunomodulatory functions (Hernández Figueroa et al., 2024; Yao et al., 2025). Similarly, benzoic acid and 3-(4-hydroxyphenyl) lactic acid showed predicted antimicrobial and immunomodulatory activities, aligning with existing literature (Rajanikar et al., 2021; Chen et al., 2024).

PASS-based cytotoxicity predictions suggested potential anticancer activity for benzoic acid and 3-(4-hydroxyphenyl)lactic acid, with predicted efficacy against selected cancer cell lines (Table 5). However, these predictions should be interpreted cautiously, as they are highly dependent on compound concentration, cellular context, and experimental conditions. While PLA lacked direct cytotoxicity prediction outputs, previous studies suggest its involvement in immune modulation and tumor microenvironment regulation (Kim et al., 2024). Overall, these *in silico* analyses provide a framework for prioritizing metabolites for targeted experimental investigation rather than definitive evidence of therapeutic efficacy. Taken together, the predicted low toxicity, favorable ADME parameters, and putative bioactivities of Gt28L-derived metabolites support their potential relevance in probiotic and postbiotic formulations. However, these findings should be regarded as hypothesis-generating and require validation through targeted *in vitro* functional assays, transcriptomic analyses, and *in vivo* studies to establish bioavailability, efficacy, and safety under physiological conditions.

**TABLE 5:** *In silico* prediction of cytotoxicity for tumor cell lines.

Metabolite	Pa	Pi	Cell-line	Cell-line name	Tissue/organ
Benzoic acid	0.869	0.003	Hs 683	Oligodendroglioma	Brain
	0.643	0.014	NCI-H838	Non-small cell lung cancer. 3 stage	Lung
Phenyllactic acid	–	–	–	No data	–
3-(4-hydroxyphenyl) lactic Acid	0.515	0.053	Hs 683	Oligodendroglioma	Brain

Pa, probability of activity; Pi, probability of inactivity. Pa and Pi values were generated using *in silico* cytotoxicity prediction models. Higher Pa values (>0.7) indicate a greater predicted likelihood of cytotoxic activity against the indicated cell line. “–” indicates data not available.

### 3.7 Chemical characterization of extracellular metabolites

**FIGURE 7:**
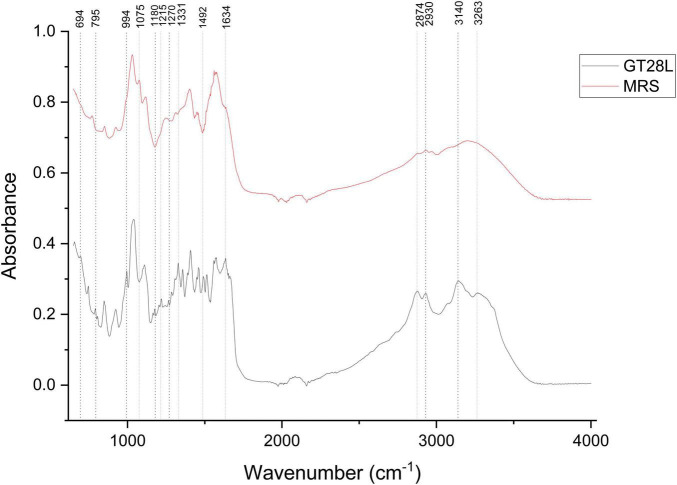
FTIR spectra of cell-free supernatant of Gt28L.

The FTIR spectra of the extracellular metabolites produced by the Gt28L strain (Figure 7) were significantly different from the uninoculated and freeze-dried MRS medium in certain spectral regions, demonstrating biochemical changes caused by fermentation.

The stretching vibrations of carbon are very prominent in the vibrational spectrum of benzene and its derivatives; therefore, in the present study, the doubly degenerate e1u ring stretching mode (1,493 cm^–1^) and the non-degenerate a1g mode of benzene (998 cm^–1^) were attributed to C–C skeletal vibrations. The FTIR spectrum of the Gt28L sample highlights the presence of several functional group’s characteristic of extracellularly secreted secondary metabolites. The analysis was performed in the range of 600–4,000 cm^–1^, and the main absorption bands identified as different from the uninoculated MRS medium are highlighted in the range of 698–851 cm^–1^ and are specific to out-of-plane C–H deformations of the aromatic nucleus (Olsztyñska-Janus and Komorowska, 2012), indicating the presence of phenolic and aromatic compounds (e.g., phenolic acids, stilbenes, tannins). At the wavenumber 795 cm^–1^, the Ring A torsion and C–H aromatic bend of pyrocatechol, flavone, flavanol and epicatechin could be attributed (Hossain et al., 2022), which confirms the presence of catechin and epicatechin. The stretching vibrations of carbon were very prominent in the vibrational spectrum of benzene and its derivatives, so in the present study, the *e1u* ring stretching mode (1,492 cm^–1^) and the *a1g* mode of benzene (994 cm^–1^) were attributed to C–C skeletal vibrations (Sajan et al., 2006). From Figure 7, a series of specific bands of Gt28L can be observed in the range of 1,112–1,359 cm^–1^, bands associated with C–O–C, C–N bonds, and O–H vibrations, reflecting the possible presence of polysaccharide compounds, residual sugars, and/or secondary amines (Andreeva and Burkova, 2017). The range 1,462–1,577 cm^–1^ was attributed to Amide II; specific bands were found in both non-inoculated MRS and Gt28L, but the bands at 1,493 cm^–1^ and 1,516 cm^–1^ are specific to the extracellular metabolites of LAB and are attributed to C = C (aromatic) vibrations and NH (amide II) deformations, associated with phenolic compounds and microbial peptides (Adt et al., 2006). The range 1,637–1,665 cm^–1^ is specific to Amide I, the typical band for C = O stretching (amide I), highlighting protein content (possible extracellular peptides) and is found in both spectra (Ji et al., 2020). The peak identified only for Gt28L at 1,665 cm^–1^ is attributed to C = O stretch vibrations specific to flavonoids (Rocha et al., 2021). The bands at 2,874 and 2,930 cm^–1^ are due to the stretching of methylene groups (CH_2_, CH_3_), corresponding to aliphatic compounds, potentially fatty acids or biosurfactants secreted by bacteria (Dutta et al., 2013). The broad band between 3,140 and 3,263 cm^–1^ was associated with the stretching of O–H and N–H, indicating a high content of bound water, hydroxyl groups, or proteins/polysaccharides.

The Gt28L sample showed new or amplified signals in the aromatic and carbonyl compounds typical areas. The existence of these bands in the fermented sample, as well as their absence in the medium, provides evidence that LAB secretes secondary metabolites extracellularly. FTIR spectroscopy indicates that the Gt28L sample contains a diverse spectrum of bioactive components, including phenolic compounds, proteins, polysaccharides, and organic acids.

### 3.8 Antioxidant activity

**FIGURE 8:**
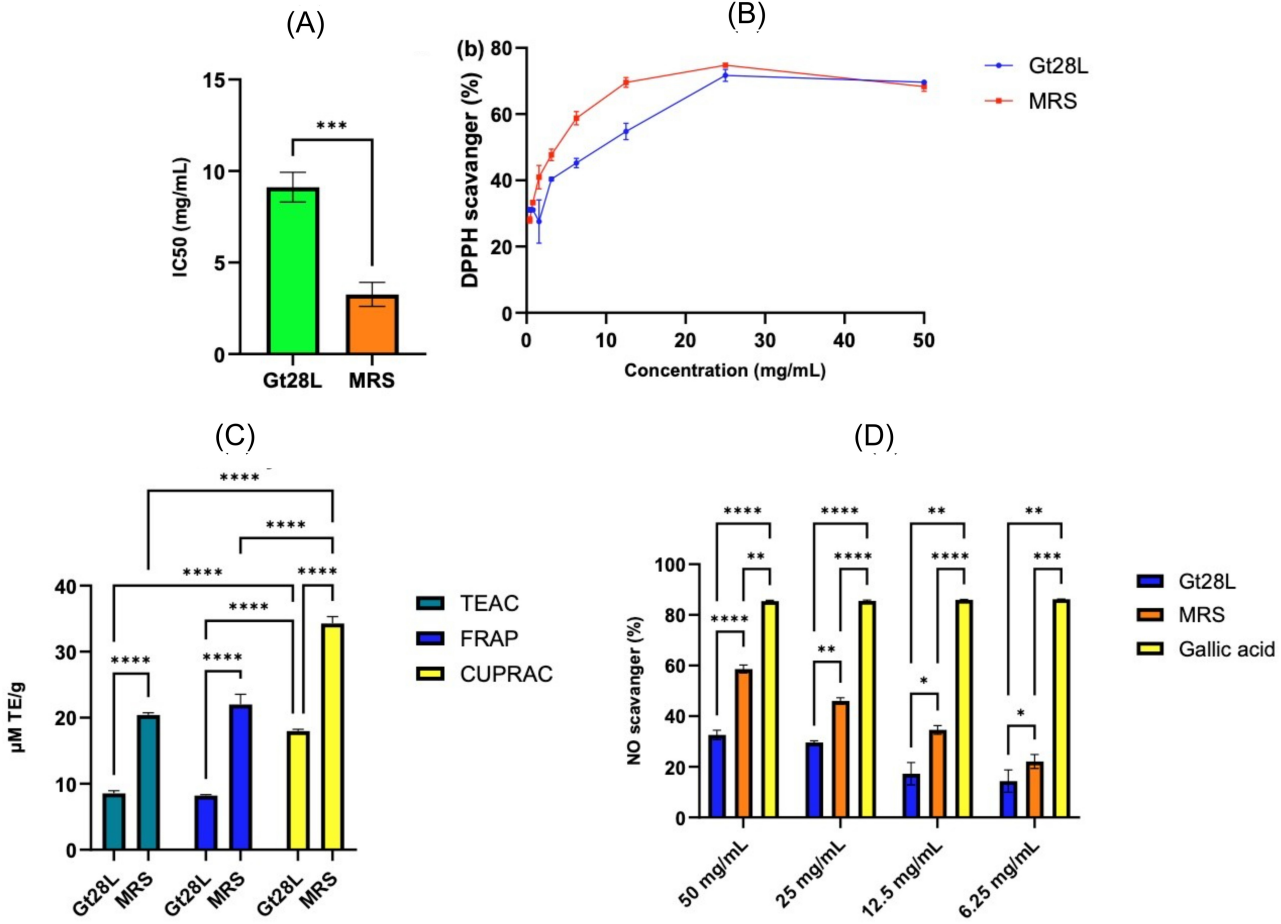
Antioxidant and nitric oxide (NO) scavenging activities of Gt28L CFS by multiple assays. **(A,B)** DPPH radical scavenging capacity and IC50 values. **(C)** Total antioxidant activity assessed by TEAC, FRAP, and CUPRAC assays. **(D)** NO scavenging activity. Data are mean ± SD (*n* = 3); *p* < 0.05 was considered significant (^****^p < 0.0001, ^***^p < 0.001, ^**^p < 0.01, *p < 0.05).

The antioxidant activity of LAB-produced secondary metabolites opens fresh opportunities in biotechnology, nutrition, and medicine, providing novel sources of bioactive chemicals with significant implications for human health and industrial sustainability. At the same time, LAB can metabolize or inactivate the existing antioxidants during growth (Rusu et al., 2023; Chandra et al., 2020; Kumano, 2024), as also suggested by our results. Gt28L CFS had significantly higher IC50 values (Figure 8A) compared to MRS broth (*p* < 0.001), implying a weaker capacity to neutralize free radicals, correlated with a reduced amount of effective phenolic antioxidants. The Shapiro-Wilk test showed that the data had a normal distribution (*W* = 0.8181, *p* = 0.0850), meeting the normality criterion at a significance level of α = 0.05. Figure 8B presents the concentration-dependent antioxidant activity for both samples, with significantly higher values of DPPH radical scavenging capacity in the case of MRS compared to Gt28L, suggesting again a higher content of antioxidants or the presence of more efficient ones in the uninoculated culture medium.

Using the FRAP, CUPRAC, and ABTS methods (Figure 8C), the antioxidant activity expressed in μM Trolox equivalents per gram of freeze-dried sample was significantly lower than that of MRS (*p* < 0.0001), indicating a lower electron transport. This difference could be also the result of the consumption of antioxidant compounds initially present in MRS correlated with a limited production of new antioxidants, particularly flavonoids or compounds with reduced groups, in the post-fermentation supernatant. The CUPRAC method was the most efficient in quantifying antioxidants (*p* < 0.0001), suggesting a predominance of compounds capable of electron transfer, specific to polyphenols, as well as of some non-phenolic compounds such as antioxidant peptides or compounds with carbonyl/conjugated groups. The FRAP and TEAC assay results were marginally lower, indicating a reduced amount of chemicals that operate by electron donation with a lower redox potential. The ANOVA conditions were satisfied since the data was normally distributed (Shapiro-Wilk, *p* > 0.1) and the variances were homogenous (Brown-Forsythe test, *p* > 0.05) (fFigure 8C).

Nitric oxide (NO) scavengers help to maintain cardiovascular, neuronal, and cellular health by lowering oxidative stress and inflammation. In general, natural antioxidants decrease excess NO by limiting the formation of reactive nitrogen species (RNS), lowering oxidative stress, inflammation, and cellular damage. This impact is critical for preventing chronic illnesses and maintaining good health (Forman and Zhang, 2021; Mladenov et al., 2023). The NO radical scavenging activity of the CFS from Gt28L and of the MRS growth medium was significantly lower than that of gallic acid, which was used as a reference antioxidant (*p* < 0.01 for all tested dosages), as shown in Figure 8D. The NO scavenging activity of all three samples varied with concentration. Gallic acid had the strongest radical scavenging activity (85.49 ± 0.29% at 50 mg/mL), followed by MRS (58.62 ± 1.60%) and Gt28L (32.60 ± 1.90%). However, the differences between MRS and Gt28L remained significant (*p* < 0.05, *p* < 0.1). While both MRS and Gt28L have some antioxidant activity, MRS outperforms Gt28L at all tested doses. This suggests that LAB secondary metabolism during fermentation may diminish the total antioxidant capacity by degrading or modifying some of the phenolic or antioxidant compounds found in MRS. These results suggest moderate anti-inflammatory and antioxidant activities. However, the presence of other bacterial secondary metabolites may provide additional functional or probiotic effects beneficial in the development of supplements or functional food.

### 3.9 Hemolytic and erythrocyte protection activity

**FIGURE 9:**
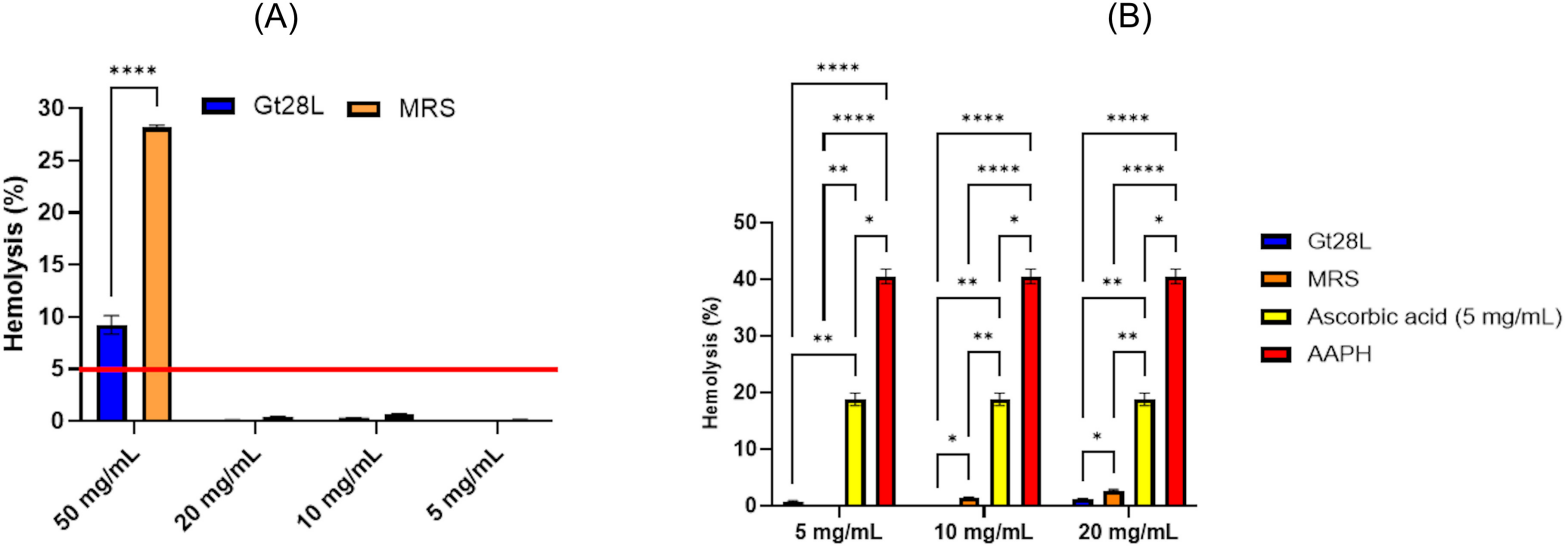
Assessment of hemolytic activity **(A)** and protection of erythrocytes against oxidative hemolysis induced by AAPH **(B)** of cell-free supernatants. RM two-way ANOVA test with the Geisser-Greenhouse correction, *n* = 3 replicates (*p < 0.05, **p < 0.01, ****p < 0.0001).

The hemocompatibility of Gt28L-derived metabolites was evaluated through hemolytic and anti-hemolytic assays using sheep erythrocytes. According to ISO 10993-4: ISO, 2017, a hemolysis index below 5% denotes acceptable blood compatibility. At the highest tested concentration (50 mg/mL), both the Gt28L and MRS samples exceeded this threshold; however, Gt28L displayed a significantly lower hemolysis rate (9.2 ± 0.9%) compared with the MRS control (28.2 ± 0.2%, *p* < 0.0001). At concentrations ≤ 20 mg/mL, the hemolysis level for Gt28L dropped below 5%, confirming its non-hemolytic and biocompatible profile (Figure 9A). The higher activity observed for MRS medium is consistent with the presence of Tween 80, a known membrane-disrupting surfactant (Muhialdin et al., 2021; Reffai and Fechtali, 2025). The anti-hemolytic potential was assessed against AAPH-induced oxidative damage. Both Gt28L and MRS significantly reduced erythrocyte lysis, achieving > 95% protection at 5–20 mg/mL, surpassing ascorbic acid (18.8 ± 1.1% hemolysis at 5 mg/mL, *p* < 0.01). The protective effect of Gt28L was concentration-dependent and slightly superior to MRS at all tested doses (*p* < 0.05). This activity likely reflects the presence of extracellular phenolic and peptide compounds with antioxidant and membrane-stabilizing functions.

The good hemocompatibility of the Gt28L sample, despite its moderate antioxidant activity, suggests that its fermented metabolites could be safely incorporated into systemic formulations, such as functional foods or dietary supplements, that require hematological safety. Even in trace amounts, flavonoids, phenolic acids, and stilbenes likely reflect cytoprotective properties (Kaźmierczak et al., 2025; Shevchenko, 2024). This effect is manifested by inhibiting membrane lipid oxidation, reducing oxidative stress, and stabilizing the structure and integrity of the cell membrane. The anti-hemolytic effect of the post-fermentation supernatant (Gt28L), MRS medium, and ascorbic acid was assessed by evaluating their capacity to protect erythrocytes against oxidative hemolysis produced by the peroxyl radical generator AAPH. Figure 9B indicates that, at all tested concentrations, AAPH generated considerable hemolysis (> 40%), demonstrating its oxidizing effect. In contrast, hemolysis was significantly decreased when erythrocytes were pre-treated with Gt28L, MRS, or ascorbic acid. The samples Gt28L and MRS significantly reduced hemolysis and offered better antioxidant protection (<3% hemolysis) than ascorbic acid at 5 mg/mL (18.80 ± 1.10% hemolysis) (*p* < 0.01). The differences between Gt28L and MRS were significant (*p* < 0.05) at all doses tested, with Gt28L offering better protection. Thus, by markedly reducing AAPH-induced oxidative stress, Gt28L exhibited notable membrane-protective properties. This protective effect is likely associated with the synthesis of extracellular phenolic metabolites possessing antioxidant activity (e.g., phenolic acids, stilbenes, flavonoids) (Rajashekar, 2023). The general trend was a decrease in hemolysis with the decrease of the sample concentration, suggesting that at higher doses, certain components may exhibit a pro-oxidative effect. Antioxidant compounds may exhibit concentration-dependent dual behavior, acting as pro-oxidants at elevated concentrations and thereby exacerbating oxidative stress rather than attenuating it (Manful et al., 2025). Consequently, the assessment of subhemolytic concentrations is critical to discriminate genuine cytoprotective effects from dose-dependent pro-oxidant activity. The CFS of Gt28L is intended for functional food and postbiotic applications, where its biological activity is expected to exert localized, transient effects within the gastrointestinal tract. Accordingly, the concentrations evaluated in this study are appropriate for assessing safety and functional efficacy under conditions that approximate physiological exposure during gastrointestinal transit, supporting their relevance for *in vitro* safety and bioactivity assessment.

## 4 Conclusion

*L. plantarum* Gt28L is a genetically stable and metabolically versatile strain with strong probiotic and postbiotic potential. Genomic analyses indicate the absence of virulence and antibiotic resistance genes, supporting its safety for human and food applications. Functional predictions and metabolomic profiling reveal production of bioactive compounds, including GABA, phenyllactic acid, and diverse amino acids, coupled with antimicrobial bacteriocins, highlighting both probiotic (live strain) and postbiotic (metabolite-mediated) functionalities. *In vitro* cytocompatibility and hemolysis assays further confirm its biological safety. Future studies, including *in vivo* validation and integrative transcriptomic and metabolomic analyses, are needed to confirm functional efficacy, host interaction, and clinical potential. Overall, Gt28L represents a promising candidate for food, health, and biotechnological applications.

## Data Availability

The genome assembly data of Gt28L have been deposited in the NCBI Sequence Read Archive under BioProject ID PRJNA1116628 (https://www.ncbi.nlm.nih.gov/sra/PRJNA1116628) and BioSample accession SAMN49560224.

## Footnotes

1
http://gctype.wdcm.org

2
https://github.com/sanger-pathogens/Roary

3
http://www.swissadme.ch/

4
https://comptox.charite.de/protox3/

5
https://www.way2drug.com/passonline/
