# Supplementary material for: Genomic and metabolomic insights into the antimicrobial and therapeutic potential of Lactiplantibacillus plantarum UTNGt28L isolated from Amazonian star apple
Source: Front Microbiol. 2026 Feb 18;17:1771106. doi: 10.3389/fmicb.2026.1771106 (PMC12957097; doi:10.3389/fmicb.2026.1771106)
Supplement: Supplementary file 1 [file Data_Sheet_1.docx]

**Supplementary files**

**Genomic and Metabolomic Insights into the Antimicrobial, Functional, and Therapeutic Potential of *Lactiplantibacillus plantarum* UTNGt28L isolated from Amazonian star apple**

Gabriela N. Tenea^*1^, Ioana C. Marinas^2^, Gratiela Gradisteanu Pircalabioru^2^, Jazmin Hidalgo^1^, Mariana C. Chifiriuc^2^, Mayte Noboa^1^

^1^Biofood and Nutraceutics Research and Development Group; Faculty of Engineering in Agricultural and Environmental Sciences, Universidad Técnica del Norte, Ibarra, Ecuador.

^2^Research Institute of the University of Bucharest—ICUB, University of Bucharest, 91-95 Splaiul Independentei St., 5050095 Bucharest, Romania

**Table S1.** Assembly summary of contigs

| **Sample** | **Contigs** | **Total contig bases** | **N50** | **Max length** | **Min length** | **Mean length** |
| --- | --- | --- | --- | --- | --- | --- |
| UTNGt28L | 12 | 3,231,781 | 662,366 | 830,264 | 1,329 | 269,315 |

Contigs: The number of contigs assembled

Total bases of contigs : The total length of contigs

N50: 50% of all contig bases come from contigs longer than this value

Max length: The length of the longest contig

Min length: The length of the shortest contig

Mean length: The average length of contigs assembled

**Table S2.** Mapping overall results

| **Library name** | **Total reads** | **Mapped reads** | **Coverage (%)** | **Depth** | **Ins.size (Std.)** |
| --- | --- | --- | --- | --- | --- |
| UTNGt28L | 4,542,808 | 4,539,714 (99.93%) | 99.99 | 208.22 | 421.14 (99.48) |

Library name : Sample’s library name

Total reads : Total number of reads

Mapped reads : Total number of mapped reads

Coverage (%) : The percentage of mapped sited (>= 1x)

Depth : Average mapping depth

Ins.size (Std.) : The length between adapters and standard deviation of predicted length

**Table S3.** Busco analysis result

*Used Lineage : bacteria_odb10 (number of genomes: 4085, number of BUSCOs: 124)*

| **Status** | **# of BUSCOs** | **Percentage (%)** |
| --- | --- | --- |
| Complete BUSCOs (C) |  | |
| Complete and single-copy BUSCOs (S) | 123 | 99.19 |
| Complete and duplicated BUSCOs (D) | 1 | 0.81 |
| Fragmented BUSCOs (F) | 0 | 0.00 |
| Missing BUSCOs (M) | 0 | 0.00 |
| Total BUSCO groups searched | 124 | 100 |

Status : A quantitative assessment list of the completeness in terms of expected gene content.

The following two conditions are used to create a status:

a. Expected range of scores

b. Expected range of length alignments

If both conditions are met, it is classified as Complete (These complete busco matches are

either single-copy or duplicated). If length alignments is not met, it is classified as Fragmented.

If both conditions are not met, it is classified as Missing.

# of BUSCOs : Identified count in sample

Percentage : Identified percentage in sample

**Table S4.** List of strains used in pangenome analysis

| **Assembly** | **strain** |
| --- | --- |
| ASM302883v1 | [*Lactobacillus sp. D1501*](https://www.ncbi.nlm.nih.gov/taxonomy/2108362) |
| [ASM200538v2](https://www.ncbi.nlm.nih.gov/datasets/genome/GCF_002005385.2/) | *Lactiplantibacillus plantarum 10CH* |
| [ASM3143295v1](https://www.ncbi.nlm.nih.gov/datasets/genome/GCF_031432955.1/) | *Lacticaseibacillus plantarum WCFS1* |
| [ASM1413173v1](https://www.ncbi.nlm.nih.gov/datasets/genome/GCF_014131735.1/) | *Lactiplantibacillus plantarum kckm 0112* |
| [ASM364118v1](https://www.ncbi.nlm.nih.gov/datasets/genome/GCF_003641185.1/) | *Lactiplantibacillus plantarum DSM20314* |
| [ASM1348780v1](https://www.ncbi.nlm.nih.gov/datasets/genome/GCF_013487805.1/) | *Lactiplantibacillus plantarum bk 021* |
| [ASM20385v3](https://www.ncbi.nlm.nih.gov/datasets/genome/GCF_000203855.3/) | *Lactiplantibacillus plantarum wcfs1* |
| [ASM334537v1](https://www.ncbi.nlm.nih.gov/datasets/genome/GCF_003345375.1/) | *Lactiplantibacillus plantarum TMW 1.1478* |
| [ASM2886944v1](https://www.ncbi.nlm.nih.gov/datasets/genome/GCF_028869445.1/) | *Lactiplantibacillus plantarum VHProbi V22* |

**Table S5.** Antibiotic resistance genes predicted in the UTNGt28L genome with CARD database.

| **Annotated Gene number** | **Prevalence values (%)** | **Prevalence sequence ID- microorganisms-gene name** |
| --- | --- | --- |
| gene00060 | 42.7 | ID:274702 \| Staphylococcus aureus \| ID:274702 \| ARO_Name:mgrA \| ARO:3000815 |
| gene00213 | 56.4 | ID:274693 \| Staphylococcus aureus \| ID:274693 \| ARO_Name:arlR \| ARO:3000838 |
| gene00329 | 47 | ID:46451 \| Providencia stuartii \| ID:46451 \| ARO_Name:Ecol_fabI_MULT \| ARO:3004045 |
| gene00387 | 43.6 | ID:264556 \| Streptococcus pneumoniae \| ID:264556 \| ARO_Name:Spne_PBP1a_AMX \| ARO:3003041 |
| gene00460 | 54 | ID:264281 \| Streptococcus pneumoniae \| ID:264281 \| ARO_Name:Spne_parC_FLO \| ARO:3003311 |
| gene00461 | 67.4 | ID:200134 \| Staphylococcus aureus \| ID:200134 \| ARO_Name:Saur_parE_FLO \| ARO:3003315 |
| gene00483 | 43.3 | ID:263435 \| Enterococcus faecalis \| ID:263435 \| ARO_Name:dfrE \| ARO:3002875 |
| gene00484 | 53.1 | ID:174714 \| Mycobacterium tuberculosis \| ID:174714 \| ARO_Name:Mtub_thyA_PAS \| ARO:3004153 |
| gene00633 | 43.5 | ID:39063 \| Enterococcus faecium \| ID:39063 \| ARO_Name:vanH_in_vanM_cl \| ARO:3002947 |
| gene00685 | 73 | ID:2029 \| Staphylococcus aureus \| ID:2029 \| ARO_Name:Ecol_EFTu_PLV \| ARO:3003369 |
| gene00741 | 54.5 | ID:268673 \| Staphylococcus aureus \| ID:268673 \| ARO_Name:Saur_ileS_MUP \| ARO:3003729 |
| gene00805 | 57.3 | ID:263577 \| Staphylococcus arlettae \| ID:263577 \| ARO_Name:vanT_in_vanG_cl \| ARO:3002972 |
| gene00816 | 45.3 | ID:275006 \| Staphylococcus aureus \| ID:275006 \| ARO_Name:Saur_murA_FOF \| ARO:3003776 |
| gene00996 | 45.5 | ID:272082 \| Staphylococcus aureus \| ID:272082 \| ARO_Name:arlR \| ARO:3000838 |
| gene01115 | 47.6 | ID:42499 \| Shigella sonnei \| ID:42499 \| ARO_Name:mdtG \| ARO:3001329 |
| gene01160 | 52 | ID:236 \| Bacteroides fragilis \| ID:236 \| ARO_Name:tet(Q) \| ARO:3000191 |
| gene01200 | 40.6 | ID:275386 \| Staphylococcus aureus \| ID:275386 \| ARO_Name:Saur_walK_DAP \| ARO:3003794 |
| gene01201 | 47.3 | ID:152088 \| Klebsiella pneumoniae \| ID:152088 \| ARO_Name:vanR_in_vanM_cl \| ARO:3002928 |
| gene01228 | 56.1 | ID:245722 \| Staphylococcus aureus \| ID:245722 \| ARO_Name:Saur_gyrA_FLO \| ARO:3003296 |
| gene01229 | 66.7 | ID:202688 \| Staphylococcus aureus \| ID:202688 \| ARO_Name:Saur_gyrB_AMU \| ARO:3003301 |
| gene01417 | 41.4 | ID:170125 \| Escherichia marmotae \| ID:170125 \| ARO_Name:bacA \| ARO:3002986 |
| gene01428 | 48 | ID:42638 \| Shigella sonnei \| ID:42638 \| ARO_Name:mdtG \| ARO:3001329 |
| gene01433 | 42.1 | ID:36145 \| Bacillus subtilis \| ID:36145 \| ARO_Name:lmrB \| ARO:3002813 |
| gene01470 | 54.1 | ID:158219 \| Staphylococcus arlettae \| ID:158219 \| ARO_Name:vanY_in_vanM_cl \| ARO:3002961 |
| gene01605 | 46.4 | ID:182338 \| Listeria monocytogenes \| ID:182338 \| ARO_Name:Lmon_mprF \| ARO:3003770 |
| gene01634 | 43.2 | ID:44383 \| Enterococcus faecalis \| ID:44383 \| ARO_Name:poxtA \| ARO:3004470 |
| gene01638 | 57.5 | ID:221878 \| Pseudomonas aeruginosa \| ID:221878 \| ARO_Name:AAC(3)-IIb \| ARO:3002534 |
| gene01649 | 42.7 | ID:268656 \| Staphylococcus aureus \| ID:268656 \| ARO_Name:arlR \| ARO:3000838 |
| gene01767 | 40.8 | ID:34377 \| Stenotrophomonas maltophilia \| ID:34377 \| ARO_Name:vanH_in_vanO_cl \| ARO:3002948 |
| gene01787 | 49 | ID:42312 \| Staphylococcus aureus \| ID:42312 \| ARO_Name:qacJ \| ARO:3007014 |
| gene01865 | 43.7 | ID:269070 \| Staphylococcus aureus \| ID:269070 \| ARO_Name:Saur_LmrS \| ARO:3004572 |
| gene01879 | 40.2 | ID:183097 \| Streptococcus mutans \| ID:183097 \| ARO_Name:vanY_in_vanG_cl \| ARO:3002959 |
| gene01885 | 70.7 | ID:37372 \| Bacillus subtilis \| ID:37372 \| ARO_Name:Bsub_rpoB_RIF \| ARO:3007073 |
| gene01888 | 67.2 | ID:210049 \| Mycobacterium kansasii \| ID:210049 \| ARO_Name:Mtub_rpsL_STR \| ARO:3003395 |
| gene01890 | 73.7 | ID:272240 \| Staphylococcus aureus \| ID:272240 \| ARO_Name:Saur_fusA_FA \| ARO:3003735 |
| gene01891 | 66.7 | ID:174210 \| Neisseria meningitidis \| ID:174210 \| ARO_Name:rpsJ \| ARO:3003930 |
| gene02133 | 41.9 | ID:270753 \| Staphylococcus aureus \| ID:270753 \| ARO_Name:Saur_LmrS \| ARO:3004572 |
| gene02292 | 53.2 | ID:205538 \| Streptococcus agalactiae \| ID:205538 \| ARO_Name:patB \| ARO:3000025 |
| gene02293 | 50.2 | ID:157832 \| Streptococcus pneumoniae \| ID:157832 \| ARO_Name:patA \| ARO:3000024 |
| gene02295 | 54.1 | ID:269411 \| Staphylococcus aureus \| ID:269411 \| ARO_Name:norC \| ARO:3007010 |
| gene02690 | 43.8 | ID:265744 \| Streptococcus agalactiae \| ID:265744 \| ARO_Name:patB \| ARO:3000025 |
| gene02695 | 51.6 | ID:173940 \| Staphylococcus hominis \| ID:173940 \| ARO_Name:Saur_pgsA_DAP \| ARO:3003323 |
| gene02735 | 42.9 | ID:190424 \| Pseudomonas synxantha \| ID:190424 \| ARO_Name:vanG \| ARO:3002909 |
| gene02748 | 60 | ID:270662 \| Staphylococcus aureus \| ID:270662 \| ARO_Name:Saur_murA_FOF \| ARO:3003776 |
| gene02773 | 41.7 | ID:264075 \| Enterococcus faecalis \| ID:264075 \| ARO_Name:efrA \| ARO:3003948 |
| gene02820 | 40.6 | ID:4448 \| Staphylococcus arlettae \| ID:4448 \| ARO_Name:qacG \| ARO:3007015 |
| gene02821 | 44.3 | ID:173754 \| Staphylococcus aureus \| ID:173754 \| ARO_Name:qacJ \| ARO:3007014 |
| gene02830 | 48.8 | ID:272301 \| Staphylococcus aureus \| ID:272301 \| ARO_Name:Saur_cls_DAP \| ARO:3003074 |
| gene02903 | 51.6 | ID:26085 \| Brevibacillus laterosporus \| ID:26085 \| ARO_Name:vanR_in_vanF_cl \| ARO:3002925 |

**Table S6.** Virulence genes predicted in the Gt28L genome.

| **Annotated gene number** | **Prevalence similarity value (%)** | **Gene name** |
| --- | --- | --- |
| gene00036 | 46.8 | VFG030686(gb\|YP_001702115) (sugC) Probable sugar ABC transporter, ATP-binding protein SugC |
| gene00073 | 50.3 | VFG015885(gb\|WP_003381688) (cysC1) adenylyl-sulfate kinase |
| gene00111 | 49 | VFG049038(gb\|WP_032448055) (KPN2242_RS16175) glycosyltransferase family 2 protein |
| gene00171 | 48.7 | VFG009599(gb\|WP_011558674) (narG) nitrate reductase subunit alpha |
| gene00172 | 55.8 | VFG022803(gb\|WP_041317387) (narH) nitrate reductase subunit beta |
| gene00211 | 63.3 | VFG048851(gb\|WP_015958700) (gndA) NADP-dependent phosphogluconate dehydrogenase |
| gene00213 | 76.8 | VFG006826(gb\|NP_464902) (lisR) two-component response regulator |
| gene00279 | 46.2 | VFG032386(gb\|WP_014093165) (stp) Stp1/IreP family PP2C-type Ser/Thr phosphatase |
| gene00282 | 40.1 | VFG046604(gb\|WP_012280804) (rpe) ribulose-phosphate 3-epimerase |
| gene00289 | 45.1 | VFG052330(gb\|WP_012231872.1) (acpP2) acyl carrier protein |
| gene00307 | 41.9 | VFG009364(gb\|WP_083045543) (trpD) anthranilate phosphoribosyltransferase |
| gene00318 | 45.3 | VFG052301(gb\|WP_011179437.1) (fabZ) 3-hydroxyacyl-ACP dehydratase FabZ |
| gene00320 | 42.6 | VFG011430(gb\|WP_002963616) (acpXL) acyl carrier protein |
| gene00322 | 41 | VFG038840(gb\|WP_005300916) (flmH) short chain dehydrogenase/reductase family oxidoreductase |
| gene00386 | 44.4 | VFG036559(gb\|WP_013449339) (fbpC) iron(III) ABC transporter, ATP-binding protein |
| gene00409 | 42.1 | VFG032493(gb\|WP_014093186) (lspA) signal peptidase II |
| gene00426 | 48.1 | VFG005195(gb\|WP_000006705) (pavA) Fibronectin-binding protein-like protein A |
| gene00474 | 50.7 | VFG006042(gb\|WP_001222601) (SAK_RS06335) LysR family transcriptional regulator |
| gene00491 | 43.2 | VFG043551(gb\|WP_010908478) (ML_RS08565) HU family DNA-binding protein |
| gene00511 | 53.2 | VFG049194(gb\|WP_004150973) (clpV) ATP-dependent chaperone ClpB |
| gene00517 | 46.4 | VFG007915(gb\|WP_011560224) (ddrA) ATP-binding cassette domain-containing protein |
| gene00526 | 50.6 | VFG005580(gb\|WP_000022813) (eno) phosphopyruvate hydratase |
| gene00566 | 60.7 | VFG026980(gb\|YP_005923858) (sigA/rpoV) RNA polymerase sigma factor |
| gene00601 | 49.2 | VFG032820(gb\|WP_041176241) (dltA) D-alanine--poly(phosphoribitol) ligase subunit DltA |
| gene00605 | 60.5 | VFG043573(gb\|NP_219906) (dnaK) chaperone protein DnaK |
| gene00626 | 50.2 | VFG002189(gb\|WP_002359680) (cpsB/cdsA) phosphatidate cytidylyltransferase |
| gene00627 | 69.7 | VFG045688(gb\|WP_002294134) (cpsA/uppS) undecaprenyl diphosphate synthase |
| gene00644 | 40.6 | VFG012939(gb\|WP_000703656) (gtrB) bactoprenol glucosyl transferase |
| gene00674 | 43.2 | VFG050210(gb\|WP_000467926) (BCAH187_RS26575) CpsD/CapB family tyrosine-protein kinase |
| gene00684 | 52 | VFG005547(gb\|WP_000107749) (tig/ropA) trigger factor |
| gene00685 | 71.2 | VFG046474(gb\|WP_014714676) (tufA) elongation factor Tu |
| gene00712 | 42.6 | VFG016506(gb\|WP_020002786) (pdhB) alpha-ketoacid dehydrogenase subunit beta |
| gene00796 | 44.8 | VFG015903(gb\|WP_011169509) (argK) ornithine carbamoyltransferase |
| gene00802 | 44 | VFG047710(gb\|WP_014547360) (carB) carbamoyl phosphate synthase large subunit |
| gene00898 | 41.1 | VFG001306(gb\|WP_000636149) (cap8J) type 8 capsular polysaccharide synthesis protein Cap8J |
| gene00914 | 41.3 | VFG030696(gb\|WP_014711073) (sugC) sn-glycerol-3-phosphate ABC transporter ATP-binding protein UgpC |
| gene00934 | 50.3 | VFG002181(gb\|WP_002387127) (cpsJ) ABC transporter, ATP-binding protein |
| gene00935 | 40.5 | VFG002180(gb\|WP_002362627) (cpsK) ABC transporter, permease protein |
| gene00951 | 53 | VFG006719(gb\|WP_003762516) (lap) Listeria adhesion protein Lap |
| gene00960 | 40.7 | VFG030679(gb\|WP_011730288) (sugC) sn-glycerol-3-phosphate ABC transporter ATP-binding protein UgpC |
| gene00990 | 44.8 | VFG016389(gb\|WP_000727070) (BCE_RS25805) LytR family transcriptional regulator |
| gene00996 | 44.4 | VFG024189(gb\|WP_008254065) (mprA) two-component system response regulator MprA |
| gene01022 | 51.1 | VFG031457(gb\|NP_216961) (ndk) nucleoside diphosphate kinase |
| gene01074 | 46.8 | VFG030724(gb\|WP_015308640) (sugC) sn-glycerol-3-phosphate ABC transporter ATP-binding protein UgpC |
| gene01196 | 52.3 | VFG005533(gb\|WP_002262650) (htrA/degP) trypsin-like peptidase domain-containing protein |
| gene01201 | 50.2 | VFG031731(gb\|WP_011558087) (regX3) two-component sensory transduction protein RegX |
| gene01255 | 58.9 | VFG031940(gb\|WP_014931476) (lap) Listeria adhesion protein Lap |
| gene01281 | 48.4 | VFG000080(gb\|NP_464522) (clpE) ATP-dependent protease |
| gene01287 | 50 | VFG037029(gb\|WP_010951343) (katA) catalase |
| gene01299 | 40.6 | VFG005069(gb\|WP_000636151) (cap8J) type 8 capsular polysaccharide synthesis protein Cap8J |
| gene01316 | 67.9 | VFG006815(gb\|WP_010959001) (bsh) bile salt hydrolase |
| gene01357 | 57.8 | VFG050191(gb\|WP_001084667) (galE) UDP-glucose 4-epimerase GalE |
| gene01488 | 40.2 | VFG007687(gb\|WP_001245629) (cpsF) exopolysaccharide biosynthesis glycosyltransferase VpsK |
| gene01623 | 57.5 | VFG050191(gb\|WP_001084667) (galE) UDP-glucose 4-epimerase GalE |
| gene01638 | 67.9 | VFG012103(gb\|WP_003514589) (groEL) chaperonin GroEL |
| gene01640 | 49 | VFG005841(gb\|WP_002262748) (SMU_RS01255) rhamnose-glucose polysaccharide biosynthesis protein RgpB |
| gene01649 | 42.8 | VFG031738(gb\|YP_001704775) (regX3) Sensory transduction protein RegX3 |
| gene01660 | 56.1 | VFG006806(gb\|NP_466005) (lgt) prolipoprotein diacylglyceryl transferase |
| gene01662 | 75.7 | VFG005871(gb\|WP_002991444) (hasC) UTP--glucose-1-phosphate uridylyltransferase HasC |
| gene01682 | 41.4 | VFG018246(gb\|WP_011261308) (luxS) S-ribosylhomocysteinase |
| gene01691 | 70.4 | VFG000077(gb\|NP_465991) (clpP) ATP-dependent Clp protease proteolytic subunit |
| gene01694 | 58.8 | VFG005360(gb\|WP_002262489) (plr/gapA) type I glyceraldehyde-3-phosphate dehydrogenase |
| gene01697 | 72 | VFG005582(gb\|WP_002897814) (eno) phosphopyruvate hydratase |
| gene01723 | 44.3 | VFG013515(gb\|WP_011961967) (mrsA/glmM) phosphoglucosamine mutase |
| gene01752 | 40.9 | VFG032991(gb\|WP_010990928) (oatA) peptidoglycan O-acetyltransferase |
| gene01872 | 41.3 | VFG016423(gb\|WP_000727056) (BALH_RS26190) LytR family transcriptional regulator |
| gene01883 | 58.1 | VFG000079(gb\|NP_463763) (clpC) endopeptidase Clp ATP-binding chain C |
| gene01946 | 50.8 | VFG019048(gb\|WP_000759089) (psaA) manganese ABC transporter, manganese-binding adhesion liprotein |
| gene02010 | 57.2 | VFG019127(gb\|WP_000723325) (wecB) UDP-N-acetylglucosamine 2-epimerase (non-hydrolyzing) |
| gene02013 | 65.2 | VFG002182(gb\|WP_002376666) (cpsI) UDP-galactopyranose mutase |
| gene02022 | 48.6 | VFG005844(gb\|WP_011681165) (STER_RS05250) polysaccharide biosynthesis C-terminal domain-containing protein |
| gene02025 | 68.8 | VFG006022(gb\|WP_002947383) (rfbB) dTDP-glucose 4,6-dehydratase |
| gene02028 | 46.3 | VFG016406(gb\|WP_000467958) (BCE_RS25890) CpsD/CapB family tyrosine-protein kinase |
| gene02029 | 40.5 | VFG016418(gb\|WP_042515428) (BT9727_RS25860) tyrosine protein phosphatase |
| gene02057 | 62.8 | VFG002182(gb\|WP_002376666) (cpsI) UDP-galactopyranose mutase |
| gene02067 | 48.7 | VFG005844(gb\|WP_011681165) (STER_RS05250) polysaccharide biosynthesis C-terminal domain-containing protein |
| gene02068 | 61.4 | VFG050185(gb\|WP_001971583) (bpsD) bacterial sugar transferase family protein |
| gene02094 | 44 | VFG006784(gb\|WP_003769807) (oppA) peptide ABC transporter substrate-binding protein |
| gene02098 | 49.2 | VFG016532(gb\|NP_975942) (oppF) oligopeptide ABC transporter permease |
| gene02101 | 62.7 | VFG000080(gb\|NP_464522) (clpE) ATP-dependent protease |
| gene02304 | 46.8 | VFG031400(gb\|WP_085979799) (ctpV) copper-translocating P-type ATPase |
| gene02346 | 45.3 | VFG013731(gb\|WP_005291888) (fagC) ABC transporter ATP-binding protein |
| gene02501 | 47.6 | VFG032200(gb\|WP_014092358) (lplA1) lipoate protein ligase |
| gene02546 | 45 | VFG047713(gb\|WP_014549144) (carB) carbamoyl phosphate synthase large subunit |
| gene02547 | 42.1 | VFG047728(gb\|WP_014547361) (carA) carbamoyl phosphate synthase small subunit |
| gene02572 | 44.8 | VFG006777(gb\|WP_011701742) (lplA1) lipoate protein ligase |
| gene02585 | 53.6 | VFG032847(gb\|WP_011703227) (gtcA) wall teichoic acid glycosylation protein GtcA |
| gene02637 | 43.5 | VFG041304(gb\|WP_010947678) (lirB) Dot/Icm type IV secretion system effector LirB |
| gene02655 | 45.1 | VFG039536(gb\|NP_820549) (CBU_1566) Coxiella Dot/Icm type IVB secretion system translocated effector |
| gene02668 | 44.9 | VFG013269(gb\|WP_010945211) (orfM) deoxyribonucleotide triphosphate pyrophosphatase |
| gene02701 | 40.8 | VFG001206(gb\|WP_002219640) (fbpC) iron(III) ABC transporter, ATP-binding protein |
| gene02766 | 50 | VFG016424(gb\|WP_000276321) (manA) mannose-6-phosphate isomerase, class I |
| gene02819 | 42.9 | VFG050095(gb\|WP_000136884) (hlyIII) hemolysin III family protein |
| gene02885 | 40 | VFG036559(gb\|WP_013449339) (fbpC) iron(III) ABC transporter, ATP-binding protein |
| gene02932 | 41.2 | VFG005320(gb\|YP_001199493) (cbpD) choline binding protein D |
| gene02951 | 40.9 | VFG031747(gb\|WP_008261205) (regX3) two-component sensory transduction protein RegX |

**Table S7 .** Summary of the biosynthetic gene clusters, type, and metabolic compounds detected with antiSMASH

| **Sample contig** | **Reference**  **MiBIG cluster** | **RiPP-Type** | **Sequence similarity (%)** | **Product** | **Compound(s)** | **Organism** |
| --- | --- | --- | --- | --- | --- | --- |
| contig 1.1 | [BGC0002404](https://mibig.secondarymetabolites.org/repository/BGC0002404/index.html#r1c1) | **T3PKS** | 32.0 | Other | falcarindiol | *Solanum lycopersicum* |
|  | [BGC0001882](https://mibig.secondarymetabolites.org/repository/BGC0001882/index.html#r1c1) |  | 24.0 | Polyketide | chrysoxanthone A, chrysoxanthone B, chrysoxanthone C | *Penicillium rubens Wisconsin 54-1255* |
|  | [BGC0000286](https://mibig.secondarymetabolites.org/repository/BGC0000286/index.html#r1c1) |  | 23.0 | Polyketide | viguiepinol | *Streptomyces sp. KO-3988* |
|  | [BGC0001387](https://mibig.secondarymetabolites.org/repository/BGC0001387/index.html#r1c1) |  | 22.0 | Other | nucleocidin | *Streptomyces calvus* |
|  | [BGC0002493](https://mibig.secondarymetabolites.org/repository/BGC0002493/index.html#r1c1) |  | 21.0 | NRP | parabactin | *Paracoccus denitrificans PD1222* |
|  | [BGC0001184](https://mibig.secondarymetabolites.org/repository/BGC0001184/index.html#r1c1) |  | 21.0 | Other | bacilysin | *Bacillus velezensis FZB42* |
|  | [BGC0000888](https://mibig.secondarymetabolites.org/repository/BGC0000888/index.html#r1c1) |  | 21.0 | Other | bacilysin | *Bacillus sp. CS93* |
|  | [BGC0001863](https://mibig.secondarymetabolites.org/repository/BGC0001863/index.html#r1c1) |  | 20.0 | RiPP, Terpene | bacillicn CER074 | *Bacillus mycoides* |
|  | [BGC0001444](https://mibig.secondarymetabolites.org/repository/BGC0001444/index.html#r1c1) |  | 20.0 | Other (shikimate derived) | caboxamycin | *Streptomyces sp. NTK 937* |
|  | [BGC0000783](https://mibig.secondarymetabolites.org/repository/BGC0000783/index.html#r1c1) |  | 19.0 | Saccharide | O-antigen | *Xanthomonas oryzae pv. Oryzae* |
| contig 2.1 | [BGC0000617](https://mibig.secondarymetabolites.org/repository/BGC0000617/index.html#r1c1) | **RIPP-like** | 22.0 | RiPP | coagulin | *Bacillus coagulans* |
|  | [BGC0002585](https://mibig.secondarymetabolites.org/repository/BGC0002585/index.html#r1c1) |  | 21.0 | Other | ubericin K | *Streptococcus uberis* |
|  | [BGC0000589](https://mibig.secondarymetabolites.org/repository/BGC0000589/index.html#r1c1) |  | 20.0 | RiPP | microcin M | *Escherichia coli Nissle 1917* |
|  | [BGC0002656](https://mibig.secondarymetabolites.org/repository/BGC0002656/index.html#r1c1) |  | 19.0 | Polyketide | oryzanaphthopyran A, oryzanaphthopyran B, oryzanaphthopyran C, oryzanthrone A, oryzanthrone B, chlororyzanthrone A, chlororyzanthrone B | *Streptacidiphilus oryzae TH49* |
|  | [BGC0000586](https://mibig.secondarymetabolites.org/repository/BGC0000586/index.html#r1c1) |  | 19.0 | RiPP | microcin E492 | *Klebsiella pneumoniae RYC492* |
|  | [BGC0002579](https://mibig.secondarymetabolites.org/repository/BGC0002579/index.html#r1c1) |  | 17.0 | RiPP | carnobacteriocin XY | *Carnobacterium maltaromaticum* |
|  | [BGC0001407](https://mibig.secondarymetabolites.org/repository/BGC0001407/index.html#r1c1) |  | 16.0 | RiPP | bicereucin | *Bacillus cereus SJ1* |
|  | [BGC0001862](https://mibig.secondarymetabolites.org/repository/BGC0001862/index.html#r1c1) |  | 16.0 | RiPP | pallidocin | *Aeribacillus pallidus* |
|  | [BGC0000590](https://mibig.secondarymetabolites.org/repository/BGC0000590/index.html#r1c1) |  | 16.0 | RiPP | microcin N | *Escherichia coli* |
|  | [BGC0000558](https://mibig.secondarymetabolites.org/repository/BGC0000558/index.html#r1c1) |  | 16.0 | RiPP | sublancin 168 | *Bacillus subtilis subsp. subtilis str. 168* |
| contig 2.2 | [BGC0001291](https://mibig.secondarymetabolites.org/repository/BGC0001291/index.html#r1c1) | **Cyclic lactone autoinducer** | 19.0 | RiPP | enterocin NKR-5-3B | *Enterococcus faecium* |
|  | [BGC0002579](https://mibig.secondarymetabolites.org/repository/BGC0002579/index.html#r1c1) |  | 15.0 | RiPP | carnobacteriocin XY | *Carnobacterium maltaromaticum* |
|  | [BGC0000540](https://mibig.secondarymetabolites.org/repository/BGC0000540/index.html#r1c1) |  | 13.0 | RiPP | paenibacillin | *Paenibacillus polymyxa OSY-DF* |
|  | [BGC0000811](https://mibig.secondarymetabolites.org/repository/BGC0000811/index.html#r1c1) |  | 12.0 | Alkaloid | fumigaclavine C | *Aspergillus fumigatus Af293* |
|  | [BGC0001573](https://mibig.secondarymetabolites.org/repository/BGC0001573/index.html#r1c1) |  | 12.0 | Alkaloid | dihydrolysergic acid | *Claviceps Africana* |
|  | [BGC0002667](https://mibig.secondarymetabolites.org/repository/BGC0002667/index.html#r1c1) |  | 12.0 | RiPP | estericin A | *Clostridium estertheticum* |
|  | [BGC0001267](https://mibig.secondarymetabolites.org/repository/BGC0001267/index.html#r1c1) |  | 12.0 | Terpene | lysergic acid, elymoclavine | *Claviceps fusiformis* |
|  | [BGC0002362](https://mibig.secondarymetabolites.org/repository/BGC0002362/index.html#r1c1) |  | 11.0 | Polyketide | loseolamycin A1, loseolamycin A2 | *Micromonospora endolithica* |
|  | [BGC0000891](https://mibig.secondarymetabolites.org/repository/BGC0000891/index.html#r1c1) |  | 10.0 | Other (Aminocoumarin) | pentabromopseudilin | *Pseudoalteromonas luteoviolacea 2ta16* |
|  | [BGC0000890](https://mibig.secondarymetabolites.org/repository/BGC0000890/index.html#r1c1) |  | 0.1 | Other (Aminocoumarin) | pentabromopseudilin | *Pseudoalteromonas phenolica O-BC30* |
| contig 7.1 | [BGC0000647](https://mibig.secondarymetabolites.org/repository/BGC0000647/index.html#r1c1) | **Terpene** | 45.0 | Terpene | carotenoid | *Rhodobacter sphaeroides* |
|  | [BGC0000648](https://mibig.secondarymetabolites.org/repository/BGC0000648/index.html#r1c1) |  | 23.0 | Terpene | carotenoid | *Myxococcus xanthus* |
|  | [BGC0000656](https://mibig.secondarymetabolites.org/repository/BGC0000656/index.html#r1c1) |  | 23.0 | Terpene | zeaxanthin | *Xanthobacter autotrophicus Py2* |
|  | [BGC0000637](https://mibig.secondarymetabolites.org/repository/BGC0000637/index.html#r1c1) |  | 23.0 | Terpene | carotenoid | *Corynebacterium glutamicum* |
|  | [BGC0000645](https://mibig.secondarymetabolites.org/repository/BGC0000645/index.html#r1c1) |  | 21.0 | Terpene | carotenoid | *Halobacillus halophilus DSM 2266* |
|  | [BGC0001227](https://mibig.secondarymetabolites.org/repository/BGC0001227/index.html#r1c1) |  | 21.0 | Terpene | isorenieratene | *Streptomyces collinus Tu 365* |
|  | [BGC0000633](https://mibig.secondarymetabolites.org/repository/BGC0000633/index.html#r1c1) |  | 21.0 | Terpene | carotenoid | *Streptomyces avermitilis* |
|  | [BGC0000636](https://mibig.secondarymetabolites.org/repository/BGC0000636/index.html#r1c1) |  | 20.0 | Terpene | carotenoid | *Brevibacterium linens* |
|  | [BGC0000640](https://mibig.secondarymetabolites.org/repository/BGC0000640/index.html#r1c1) |  | 20.0 | Terpene | carotenoid | *Enterobacteriaceae bacterium DC404* |
|  | [BGC0000630](https://mibig.secondarymetabolites.org/repository/BGC0000630/index.html#r1c1) |  | 20.0 | Terpene | (2R,3S,3'S)-2-hydroxyastaxanthin | *Paracoccus haeundaensis* |

**Table S8.** ADME properties of metabolites identified in CFS by GC–MS analysis

| **Metabolite** | **MW (g/mol)** | **nHBD** | **nHBA** | **TPSA (Å²)** | **WLOGP** | **GI Absorption** | **BBB Permeant** | **Pgp Substrate** | **Bioavailability Score** | **Lipinski Violations** | **Synthetic Accessibility** |
| --- | --- | --- | --- | --- | --- | --- | --- | --- | --- | --- | --- |
| Myo-Inositol | 180.16 | 6 | 6 | 120 | -3.72 | Low | No | No | 0.17 | 1 (WLOGP) | 2.78 |
| Valine | 117.15 | 2 | 3 | 63.3 | -1.39 | High | No | No | 0.55 | 0 | 2.25 |
| Alanine | 89.09 | 2 | 3 | 63.3 | -1.41 | High | No | No | 0.55 | 0 | 2.21 |
| Lactamide | 89.09 | 2 | 3 | 63.3 | -1.41 | High | No | No | 0.55 | 0 | 2.21 |
| Leucine | 131.17 | 2 | 3 | 63.3 | -1.39 | High | No | No | 0.55 | 0 | 2.32 |
| Isoleucine | 131.17 | 2 | 3 | 63.3 | -1.39 | High | No | No | 0.55 | 0 | 2.32 |
| Serine | 105.09 | 3 | 4 | 83.1 | -2.16 | High | No | No | 0.55 | 0 | 2.29 |
| Proline | 115.13 | 2 | 3 | 63.3 | -1.38 | High | No | No | 0.55 | 0 | 2.3 |
| Glycine | 75.07 | 2 | 3 | 63.3 | -1.43 | High | No | No | 0.55 | 0 | 2.2 |
| Aspartic Acid | 133.1 | 3 | 5 | 101.3 | -2.77 | Low | No | No | 0.17 | 0 | 2.35 |
| Pyroglutamic Acid | 129.11 | 2 | 3 | 63.3 | -1.41 | High | No | No | 0.55 | 0 | 2.31 |
| Glutamic Acid | 147.13 | 3 | 5 | 101.3 | -2.77 | Low | No | No | 0.17 | 0 | 2.36 |
| 4-Aminobutanoic Acid (GABA) | 103.12 | 2 | 3 | 63.3 | -1.41 | High | No | No | 0.55 | 0 | 2.28 |
| Phenylalanine | 165.19 | 2 | 3 | 63.3 | -0.38 | High | No | No | 0.55 | 0 | 2.45 |
| Benzoic Acid | 122.12 | 1 | 2 | 37.3 | 1.87 | High | Yes | No | 0.55 | 0 | 2.4 |
| Glyceric Acid | 106.05 | 3 | 4 | 83.1 | -2.16 | High | No | No | 0.55 | 0 | 2.29 |
| Fructose | 180.16 | 5 | 6 | 110 | -3.72 | Low | No | No | 0.17 | 1 (WLOGP) | 2.78 |
| Glucose | 180.16 | 5 | 6 | 110 | -3.72 | Low | No | No | 0.17 | 1 (WLOGP) | 2.78 |
| Galactitol | 182.17 | 6 | 6 | 120 | -3.72 | Low | No | No | 0.17 | 1 (WLOGP) | 2.78 |
| Maltose | 342.3 | 8 | 11 | 210 | -5.46 | Low | No | No | 0.17 | 2 (WLOGP, TPSA) | 3.1 |
| 2-Hydroxybutyric Acid | 104.1 | 2 | 3 | 63.3 | -1.41 | High | No | No | 0.55 | 0 | 2.28 |
| 3-Hydroxy-3-Methylbutyric Acid | 118.13 | 2 | 3 | 63.3 | -1.39 | High | No | No | 0.55 | 0 | 2.3 |
| Hydroxyisocaproic Acid | 132.16 | 2 | 3 | 63.3 | -1.39 | High | No | No | 0.55 | 0 | 2.32 |
| 2-Hydroxy-3-Methylvaleric Acid | 132.16 | 2 | 3 | 63.3 | -1.39 | High | No | No | 0.55 | 0 | 2.32 |
| Palmitic Acid | 256.42 | 1 | 2 | 37.3 | 7.45 | Low | Yes | Yes | 0.17 | 1 (WLOGP) | 3.2 |
| Stearic Acid | 284.48 | 1 | 2 | 37.3 | 8.23 | Low | Yes | Yes | 0.17 | 1 (WLOGP) | 3.3 |
| Lactic Acid | 90.08 | 2 | 3 | 63.3 | -1.41 | High | No | No | 0.55 | 0 | 2.21 |
| Glycolic Acid | 76.05 | 2 | 3 | 63.3 | -1.43 | High | No | No | 0.55 | 0 | 2.2 |
| Dihydroxy-2-Methylpropanoic Acid | 106.05 | 3 | 4 | 83.1 | -2.16 | High | No | No | 0.55 | 0 | 2.29 |
| 2,4-Dihydroxybutanoic Acid | 120.07 | 3 | 4 | 83.1 | -2.16 | High | No | No | 0.55 | 0 | 2.29 |
| 3,4-Dihydroxybutanoic Acid | 120.07 | 3 | 4 | 83.1 | -2.16 | High | No | No | 0.55 | 0 | 2.29 |
| Malic Acid | 134.09 | 3 | 5 | 101.3 | -2.77 | Low | No | No | 0.17 | 0 | 2.35 |
| 3-Deoxy-Ribo-Hexonic Acid Lactone | 162.14 | 4 | 5 | 100 | -2.5 | Low | No | No | 0.17 | 0 | 2.5 |
| Pyruvic Acid | 88.06 | 1 | 3 | 63.3 | -1.41 | High | No | No | 0.55 | 0 | 2.21 |
| Succinic Acid | 118.09 | 2 | 4 | 83.1 | -2.16 | High | No | No | 0.55 | 0 | 2.29 |
| Glycerol-3-Phosphate | 172.07 | 3 | 6 | 109 | -2.06 | Low | No | No | 0.17 | 0 | 2.75 |
| Phenyllactic Acid | 166.17 | 2 | 3 | 57.53 | 0.99 | High | No | No | 0.55 | 0 | 2.78 |
| 3-(4-Hydroxyphenyl)Lactic Acid | 182.17 | 3 | 4 | 77.76 | 0.17 | High | No | No | 0.55 | 0 | 3.02 |
| Nicotinamide | 122.12 | 1 | 2 | 55.84 | -0.37 | High | No | No | 0.55 | 0 | 2.34 |

SA synthetic accessibility, GI gastrointestinal absorption, BBB blood–brain barrier permeant, Pgp P-glycoprotein substrate, MW molecular weight (g/mol), nHBD number of hydrogen bond donor, nHBA number of hydrogen bond acceptor, BS Bioavailability Score, TPSA topological polar surface area (Å²), WLOGP water partition coefficient, nLV number of Lipinski violation (Yes; 1 violation: MLOGP>4.15 or Yes; 0 violation).

**Table S9.** Toxicity properties of Gt28L metabolites identified by ProToX II web server

| **Compound** | **LD₅₀ (mg/kg, oral, rat)** | **GHS Class** | **Hepatotoxicity** | **Carcinogenicity** | **Immunotoxicity** | **Mutagenicity** | **Cytotoxicity** | **Clinical Toxicity** | **Nutritional Toxicity** |
| --- | --- | --- | --- | --- | --- | --- | --- | --- | --- |
| Myo-Inositol | >10,000 | 5 | No | No | No | No | No | None | None |
| Valine | >2,000 | 5 | No | No | No | No | No | None | None |
| Alanine | >5,000 | 5 | No | No | No | No | No | None | None |
| Lactamide | ~2,500 | 4 | No | No | No | No | No | Minimal | None |
| Leucine | >2,000 | 4 | No | No | No | No | No | None | None |
| Isoleucine | >2,000 | 5 | No | No | No | No | No | None | None |
| Serine | >2,000 | 5 | No | No | No | No | No | None | None |
| Proline | >2,000 | 4 | No | No | No | No | No | None | None |
| Glycine | >7,930 | 5 | No | No | No | No | No | None | None |
| Aspartic Acid | >5,000 (est.) | 5 | No | No | No | No | No | Mild renal/salivary effects (high dose) | None |
| Pyroglutamic Acid | 1000 | 4 | No | No | No | No | No | Mild toxicity at very high doses | None |
| Glutamic Acid | >5,000 | 5 | No | No | No | No | No | None | None |
| GABA | >2,000 | 5 | No | No | No | No | No | None | None |
| Phenylalanine | ~16,000 | 5 | No | No | No | No | No | Mild toxicity at very high doses | None |
| Benzoic Acid | ~1,220 | 5 | Mild (high doses) | No | No | No | Irritant | Skin/eye irritation possible | None |
| Glyceric Acid | 2450 | 4 | No | No | No | No | No | None | None |
| Fructose | >10,000 | 5 | No | No | No | No | No | GI distress at high intake | Yes (excess) |
| Glucose | >10,000 | 5 | No | No | No | No | No | None | None |
| Galactitol | 13500 | 6 | No | No | No | No | No | None | None |
| Maltose | >5,000 | 5 | No | No | No | No | No | None | None |
| 2-Hydroxybutyric Acid | >2,000 (est.) | 3 | No | No | No | No | No | None | None |
| 3-Hydroxy-3-Methylbutyric Acid | >2,000 (est.) | 3 | No | No | No | No | No | None | None |
| Hydroxyisocaproic Acid | 600 | 3 | No | No | No | No | No | None | None |
| 2-Hydroxy-3-Methylvaleric Acid | 600 | 3 | No | No | No | No | No | None | None |
| Palmitic Acid | 900 | 4 | No | No | No | No | No | Excess may affect lipid profile | Yes (obesity-related) |
| Stearic Acid | >5,000 | 5 | No | No | No | No | No | None | Minimal |
| Lactic Acid | ~3,543 | 5 | No | No | No | No | Mild | None | None |
| Glycolic Acid | ~1,950 | 5 | No | No | No | No | Yes (irritant) | Eye and skin irritant | None |
| Dihydroxy-2-Methylpropanoic Acid | 1190 | 4 | Mild (high doses) | No | No | No | No | None | None |
| 2,4-Dihydroxybutanoic Acid | 3300 | 5 | No | No | No | No | No | None | None |
| 3,4-Dihydroxybutanoic Acid | 3300 | 5 | No | No | No | No | No | None | None |
| Malic Acid | 2497 | 5 | No | No | No | No | Mild | None | None |
| 3-Deoxy-Ribo-Hexonic Acid Lactone | Limited data | Not classified | No | No | No | No | No | None | None |
| Pyruvic Acid | 200 | 3 | No | No | No | No | Mild | None | None |
| Succinic Acid | >5,000 | 5 | No | No | No | No | No | None | None |
| Glycerol-3-Phosphate | 2260 | 5 | No | No | No | No | No | None | None |
| Phenyllactic Acid | >2,000 (est.) | 4 | No | No | No | No | No | None | None |
| 3-(4-Hydroxyphenyl)Lactic Acid | 2000 | 4 | No | No | No | No | No | None | None |
| Nicotinamide | ~3,000 | 5 | No | No | No | No | No | None | None |

*LD50 Predicted Lethal Dosage

**Table S10.** Estimation of PASS outcome for Gt28L metabolites

| **Metabolite** | **SMILES** | **Pa (probable active)** | **Pi (probable inactive)** | **Activity** |
| --- | --- | --- | --- | --- |
| **Benzoic Acid** | C1=CC=C(C=C1)C(=O)O | 0,901 | 0,005 | Antieczematic |
|  |  | 0,717 | 0,002 | Antiinflammatory, intestinal |
|  |  | 0,856 | 0,009 | Antiseborrheic |
|  |  | 0,755 | 0,005 | 3-Phytase inhibitor |
|  |  | 0,891 | 0,004 | Feruloyl esterase inhibitor |
|  |  | 0,961 | 0,001 | Glutamyl endopeptidase II inhibitor |
|  |  | 0,931 | 0,001 | Glyoxylate reductase inhibitor |
|  |  | 0,924 | 0,002 | Fragilysin inhibitor |
|  |  | 0,917 | 0,003 | 2-Dehydropantoate 2-reductase inhibitor |
|  |  | 0,870 | 0,019 | Membrane integrity agonist |
|  |  | 0,802 | 0,005 | Macrophage colony stimulating factor agonist |
| **Phenyllactic Acid** | OC(Cc1ccccc1)C(=O)O | 0,831 | 0,004 | Antidiabetic |
|  |  | 0,703 | 0,045 | Antieczematic |
|  |  | 0,801 | 0,004 | Antihypoxic |
|  |  | 0,918 | 0,002 | 3-Phytase inhibitor |
|  |  | 0,909 | 0,004 | Feruloyl esterase inhibitor |
|  |  | 0,906 | 0,003 | Glutamyl endopeptidase II inhibitor |
|  |  | 0,907 | 0,001 | Glyoxylate reductase inhibitor |
|  |  | 0,888 | 0,003 | Fragilysin inhibitor |
|  |  | 0,888 | 0,003 | 2-Dehydropantoate 2-reductase inhibitor |
|  |  | 0,899 | 0,011 | Membrane integrity agonist |
|  |  | 0,861 | 0,003 | Macrophage colony stimulating factor agonist |
| **3-(4-Hydroxyphenyl)lactic Acid** | OC(Cc1ccc(O)cc1)C(=O)O | 0,765 | 0,005 | Antidiabetic |
|  |  | 0,769 | 0,005 | Antihypoxic |
|  |  | 0,861 | 0,008 | Antiseborrheic |
|  |  | 0,940 | 0,002 | 3-Phytase inhibitor |
|  |  | 0,904 | 0,004 | Feruloyl esterase inhibitor |
|  |  | 0,851 | 0,005 | Glutamyl endopeptidase II inhibitor |
|  |  | 0,829 | 0,003 | Glyoxylate reductase inhibitor |
|  |  | 0,792 | 0,008 | Fragilysin inhibitor |
|  |  | 0,900 | 0,003 | 2-Dehydropantoate 2-reductase inhibitor |
|  |  | 0,927 | 0,005 | Membrane integrity agonist |
|  |  | 0,787 | 0,005 | Macrophage colony stimulating factor agonist |

High Pa (>0.7) and low Pi (<0.05): The compound is likely to exhibit this activity.

**Figure S1.** ANI analysis. Percent identity heatmap resulted (A). Alignment coverage heatmap resulted (B). The cells in the heatmap corresponding to an ANI value of 95% and higher are stained red. This indicates that the corresponding strains belong to the same species. The dendrograms (in green; above and on the left side), which were constructed by the simple linkage of the ANIm (ANI with MUMmer) percentage identities, correspond to the results of the clustering of the ANI values between the used strains. ANI: Average Nucleotide Identity.

(A).


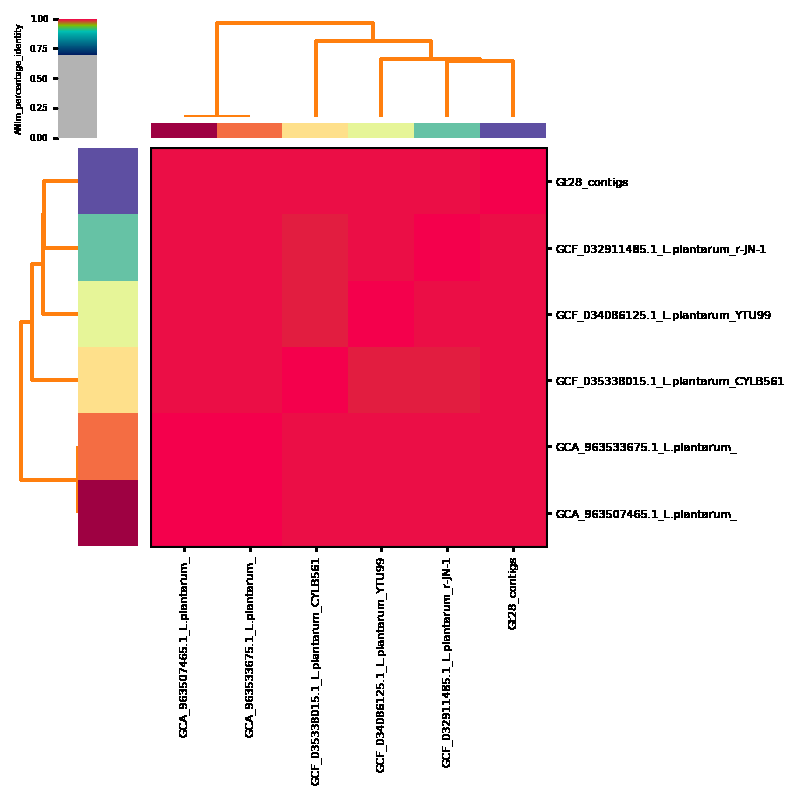


Gt28L

Gt28L

(B).


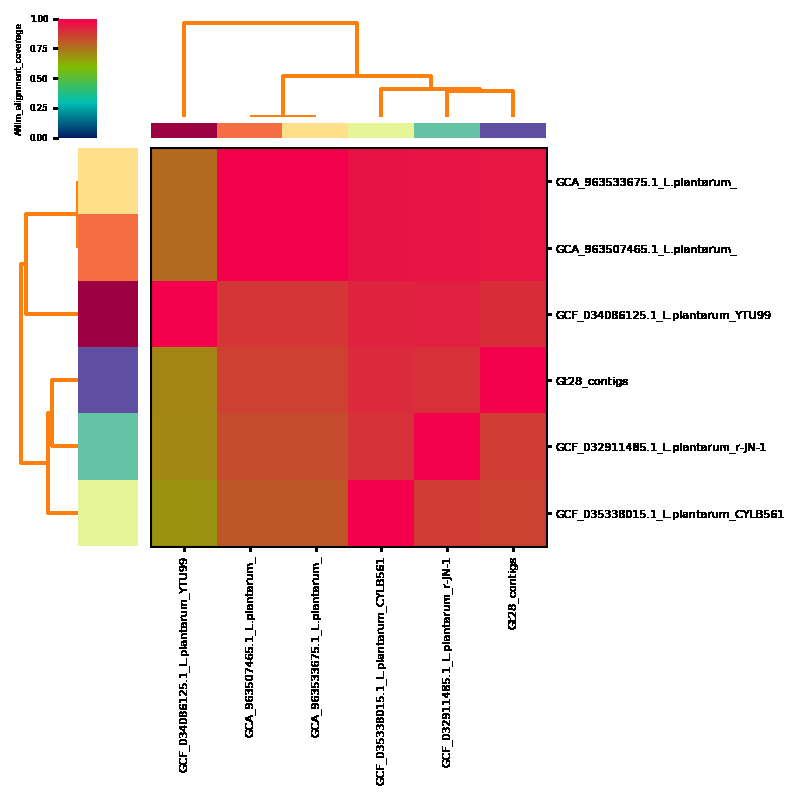


Gt28L

Gt28L

**Figure S2.** Distribution of antimicrobial resistance (AMR) features across three categories: AMR gene families, resistance mechanisms, and drug classes. The graph highlights the prevalence of specific resistance determinants, with "antibiotic target alteration" and "antibiotic efflux" being the most frequently observed mechanisms, and notable gene families such as glycopeptide resistance gene clusters and ABC efflux pumps.


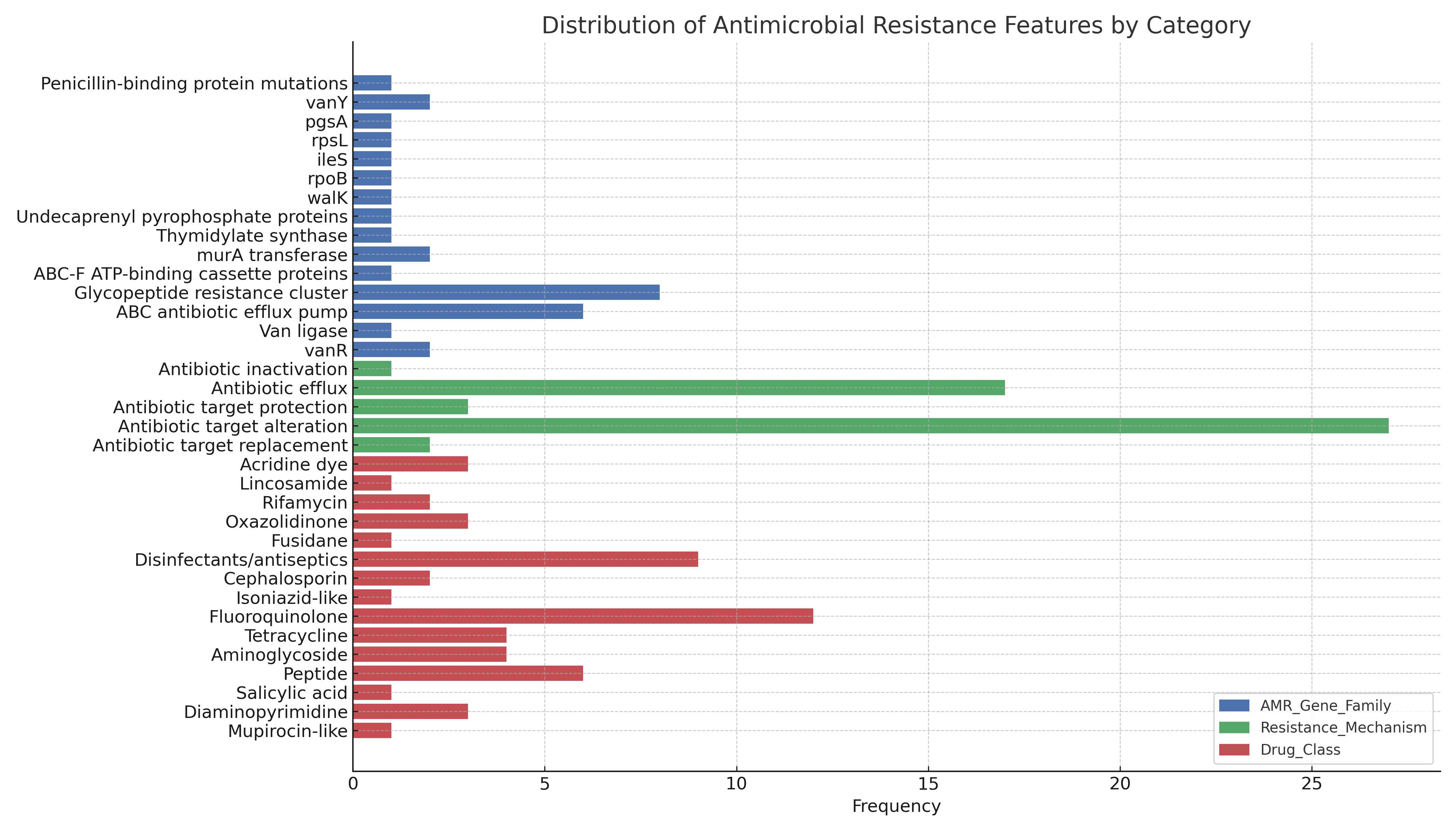


**Figure S3.** Phylogenetic placement of strain Gt28L among representative lactic acid bacteria based on whole-genome analysis. The maximum-likelihood tree was constructed using 56 conserved single-copy marker genes. Gt28L clusters closely with members of the *Lactiplantibacillus plantarum* group, particularly *L. plantarum*, *L. argentoratensis*, and *L. arizonensis*, indicating high genomic similarity. Metadata bars adjacent to each strain represent GC content, genome length, and standard deviation/mean values, supporting the genetic and structural coherence of the clade. Bootstrap support values are indicated at branching nodes.


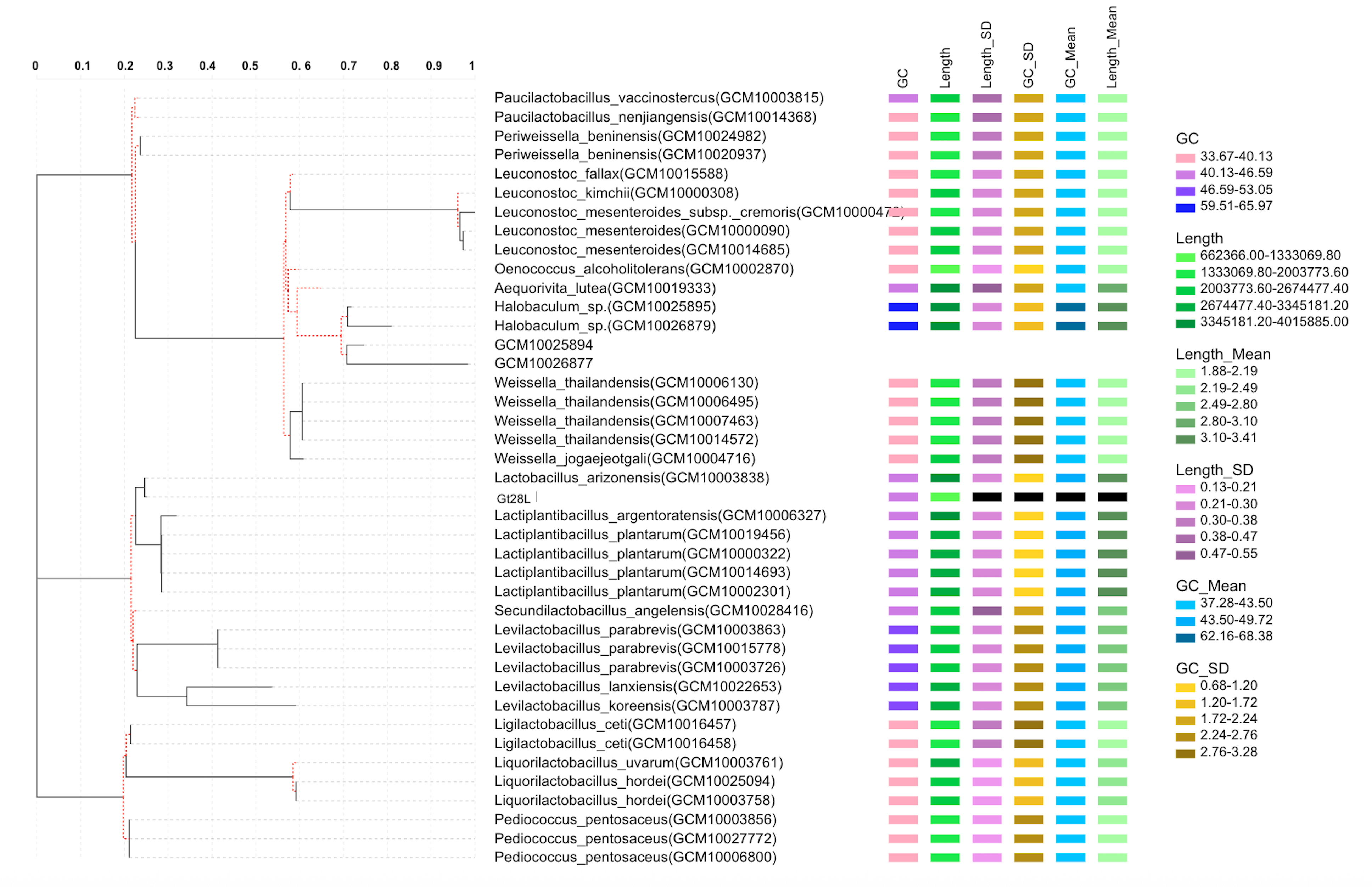


**
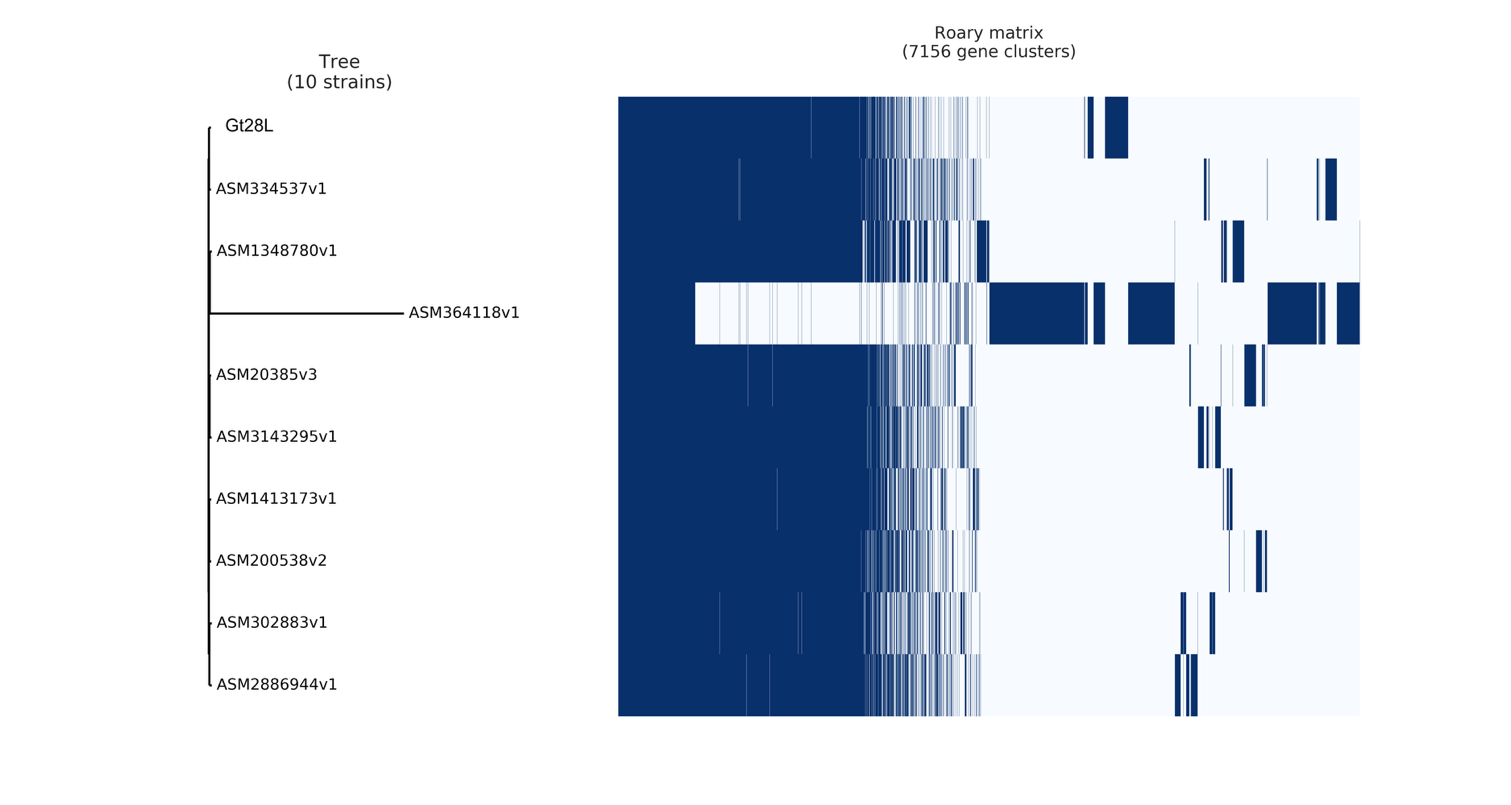
Figure S4.** Pangenome Roary matrix. Gene content comparison of the 10 considered strains. The matrix shows genes typical of each strain and those conserved in all.

**Figure S5.** Functional enrichment network of KEGG pathways in *L. plantarum* Gt28L based on metabolic genes annotation. Node size indicates the number of genes assigned to each pathway, while node color intensity reflects statistical significance (darker red indicates higher enrichment). Edges represent functional associations or shared genes between pathways.
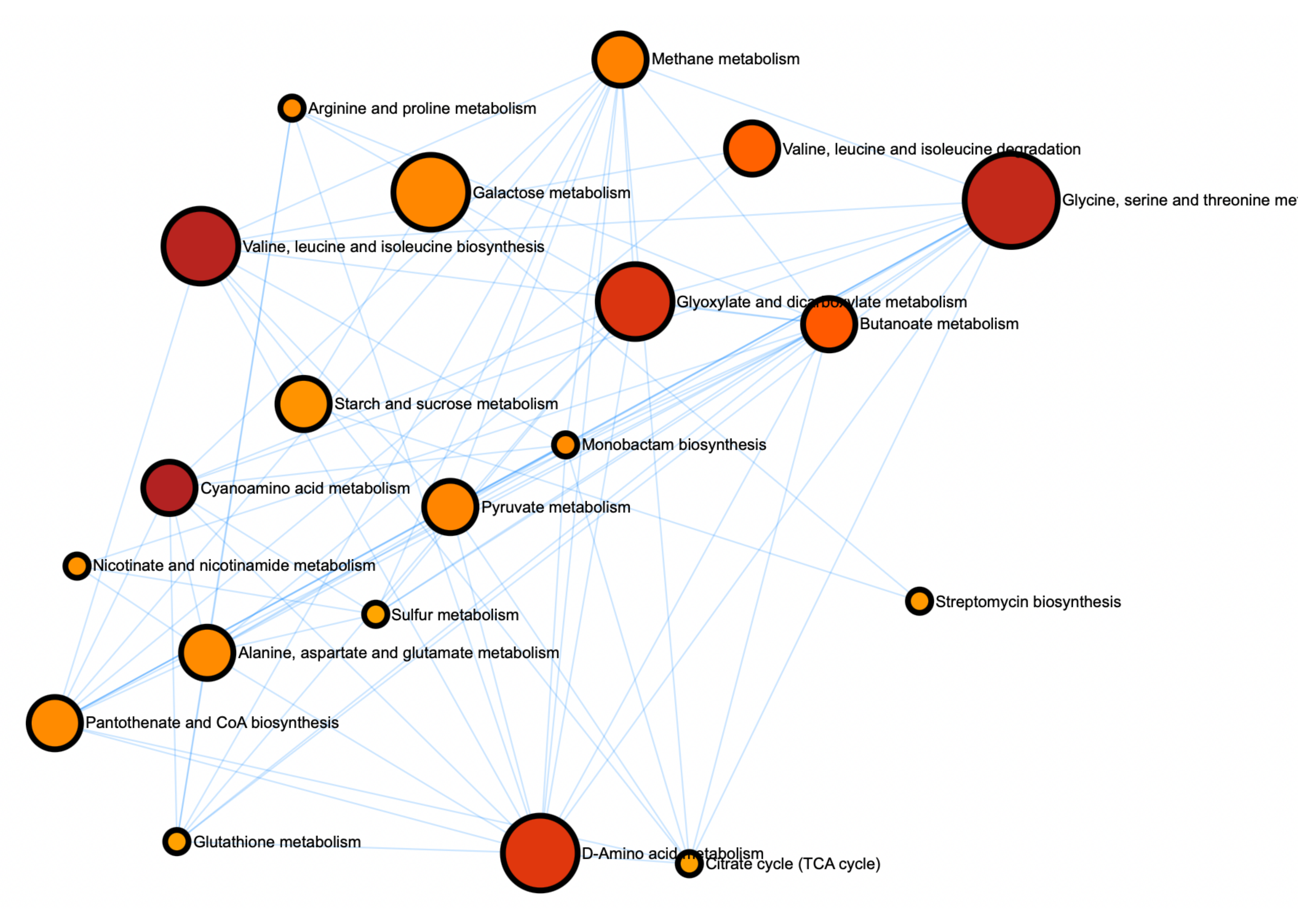


**Figure S6.** Overview of Enriched Metabolites set (top 25) **
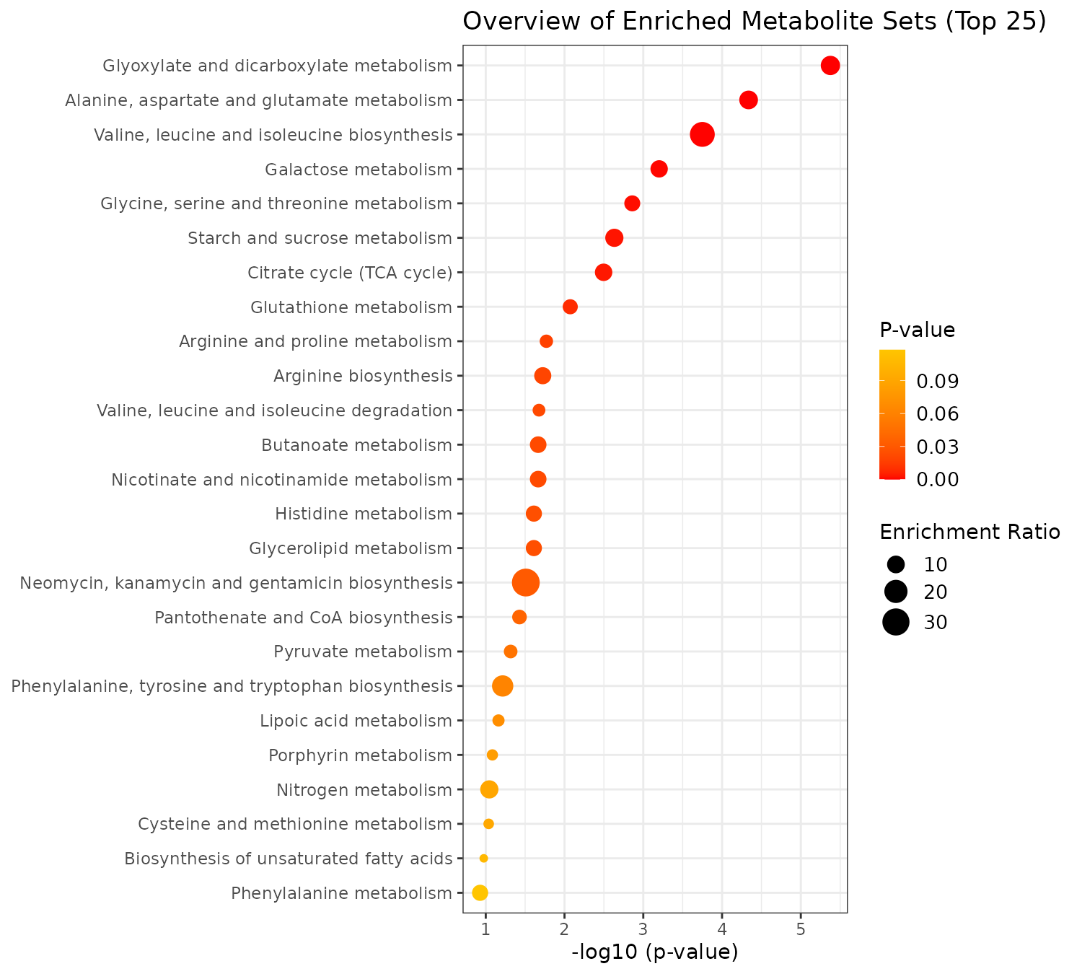
**
